# Time Course of Age-Linked Changes in Photosynthetic Efficiency of *Spirodela polyrhiza* Exposed to Cadmium

**DOI:** 10.3389/fpls.2022.872793

**Published:** 2022-05-25

**Authors:** Vesna Peršić, Jasenka Antunović Dunić, Lucija Domjan, Günther Zellnig, Vera Cesar

**Affiliations:** ^1^Department of Biology, Josip Juraj Strossmayer University of Osijek, Osijek, Croatia; ^2^Institute of Biology, University of Graz, Graz, Austria; ^3^Faculty of Dental Medicine and Health, Josip Juraj Strossmayer University of Osijek, Osijek, Croatia

**Keywords:** dose-time-age response, polyphasic chlorophyll fluorescence, photosynthesis, respiration, PCA, chloroplast ultrastructure, TEM

## Abstract

Short-term assessment of adverse effects is essential for populations exposed to higher risk of environmental pollution. This study presents the time course of physiological and morphological changes attributed to cadmium, emphasizing age-linked differences in the susceptibility of photosynthetic apparatus of *Spirodela polyrhiza* fronds exposed to different cadmium concentrations. A four-frond colony represented by mother, daughter, and granddaughter plants was exposed to cadmium concentrations for 6, 24, and 72 h to establish its effect on different generations of the great duckweed. The duration of cadmium exposure accounted for the most variation in chlorophyll content as the most influential variable, and after 72 h, frond responsiveness was a function of cadmium concentration. Carotenoid contents behaved slightly differently in fronds of different ages, with the oldest mother frond exhibiting accelerated senescence. Chlorophyll fluorescence measurements showed that cadmium affects different photosynthetic electron transport segments relative to the frond's chloroplast structure level. Photosynthesis of mother fronds exposed to low cadmium and daughter fronds exposed to high cadmium was determined by the functionality of primary electron acceptance at the PSII level. Mother plants exposed to higher cadmium concentrations were characterized by closed and inactive reaction centers, dissipated energy outflux, and inhibited photosynthesis. Young fronds exposed to low and high cadmium concentrations were characterized by increased non-reducing reaction centers and thermal phase reduction, with activated dissipative mechanisms at high cadmium concentrations. Cadmium-induced changes in the ultrastructure of chloroplasts were visible after 6 h of exposure to lowest concentrations, with gradual degradation of the thylakoid system as the fronds aged. Younger fronds responded to cadmium more dynamically through molecular, physiological, and anatomical changes and tolerated a more reduced electron transport chain under given conditions than older fronds.

## Introduction

Cadmium (Cd) is a naturally occurring heavy metal in Earth's crust, with worldwide concentrations ranging from 0.1 to 0.5 mg kg^−1^in the soil and up to 5 μg L^−1^ in soil water. Anthropogenic activities are responsible for more than 90 percent of Cd found in surface waters (UNEP, 2010). Its content in soil and waters increases nowadays because of the disposal of Cd-contaminated waste, sewage sludge, and Cd-containing fertilizers (Roberts, 2014). Cd is a non-essential metal but is persistent in the environment because of its long biological half-life, phytoaccumulation, and biomagnification, thus posing a valid public health concern (Chen et al., 2020).

Plants readily take up Cd, which then affects various metabolic activities in different tissues and cell compartments, including inhibition of photosynthesis and respiration, leading to decreased nutrient uptake, growth restraints, and accelerated senescence (Faller et al., 2005; Malec et al., 2010; Basile et al., 2015; Pietrini et al., 2016; Bora and Sarma, 2021). However, it is not always apparent whether these adverse effects are due to direct cadmium toxicity, overproduction of reactive oxygen species, Cd-induced nutrient deficiency, or all of the above. Cd is a photosynthetic inhibitor, and it affects light-harvesting complex, photosystems I and II, electron transport, synthesis of chlorophyll and carotenoids, dissociation of Rubisco subunits, induces stomatal closing, and diminishes gas exchange rates, causing damage to the function and structure of chloroplasts (Voigt and Nagel, 2002; Wang et al., 2004; Perreault et al., 2011; Garcia et al., 2018; Lysenko et al., 2020). Cd-induced response of plant metabolism depends not only on Cd concentration and exposure duration but also on plant species, their developmental stage, and environmental conditions (for detailed literature review, cf. Haider et al., 2021). Most research on Cd-induced toxicity to plants is long-term, dose-response orientated, and directed toward environmentally relevant concentrations (Tkalec et al., 2008; Chaudhuri et al., 2014; Basile et al., 2015) or alleviation effects by other compounds or microorganisms as well as remediation strategies (Guan et al., 2018; Gong et al., 2019; Huang et al., 2021). It is well-known that leaf anatomy depends on plant age, thus influencing plants' photosynthetic capacity, mitochondrial respiration, and activation of different defense mechanisms when exposed to abiotic stress (Lahive et al., 2012; Tsukaya, 2013; Moustaka et al., 2015), with young plants being more resistant to abiotic stress and older plants having less flexible adaptation mechanisms (Skórzyńska-Polit and Baszyński, 1997; Krupa and Moniak, 1998; Drazkiewicz et al., 2003). However, there is not as much research on the relationship between Cd-induced toxicity and plants' growth stages regarding the susceptibility of photosynthetic apparatus (Krupa and Moniak, 1998; Drazkiewicz et al., 2003; Fei et al., 2018; Wang et al., 2018).

*Spirodela polyrhiza* (L.) Schleiden, or great duckweed, is a common floristic element of fresh water worldwide whose associations often cover the entire water surface of slow-flowing melioration canals or standing waters surrounding agricultural areas, thus easily being exposed to Cd contamination. In general, duckweeds (Lemnaceae) are the smallest and fastest-growing angiosperms (Wang et al., 2010; Ziegler et al., 2015), and *S. polyrhiza*, with its smallest monocot genome of 158 Mb (Wang et al., 2014), is becoming a vital model plant in theoretical and applied research (Lam et al., 2014; Appenroth et al., 2015; Acosta et al., 2021). It is also becoming a crucial aquatic crop. With up to 55 tons of dry mass production per hectare per year, it has prosperous possibilities to ameliorate some current problems (Appenroth et al., 2015). *S. polyrhiza* is highly efficient in nitrogen removal, and it has been successfully used for wastewater remediation. It is also an efficient hyperaccumulator of some heavy metals (Appenroth et al., 2010; Qu et al., 2019; Singh and Malaviya, 2019). Therefore, phytoremediation and subsequent usage as livestock feed pose a specific risk factor, since other elements like heavy metals could be present in wastewater. For instance, Cd accumulation in *S. polyrhiza* is concentration- and duration-dependent (Sinha et al., 1995; Chaudhuri et al., 2014; Chen et al., 2020). It is also determined that Cd bonds to different chemical forms in duckweed roots (Xue et al., 2018) and is distributed in *S. polyrhiza* in separate fractions, i.e., 52–61 % of Cd is found in the cell wall, and 37–46 % is soluble (Su et al., 2017). Similarly, differential sensitivity to Cd and its accumulation are determined in turions and mature fronds (Srivastava and Jaiswal, 1989; Oláh et al., 2015). Furthermore, it has been determined that overexpression of plasma membrane-localized transporters *SpNramp1, SpNramp2*, and *SpNramp3* differentiates between Cd, Fe, and Mn uptake, thus enabling manipulation of Cd accumulation by transforming *S. polyrhiza* to an important transgenic enrichment line that could be used for safer phytoremediation (Chen et al., 2021).

*S. polyrhiza* has a unique plant body. A frond is a morphologically reduced leaf-like structure derived from leaves and stems. Each frond has multiple roots, and each frond is an individual plant. It is also a well-known laboratory plant because of its clonal propagation, where daughter fronds originate from the vegetative node of mother fronds and remain attached by stipule or stipe. Daughter fronds initiate new granddaughter fronds before full maturity while still attached to mother fronds. Eventually, the stipe breaks off, releasing a new colony cluster (Landolt, 1998; Lemon and Posluszny, 2000; Acosta et al., 2021). As in *Lemna* species, mother fronds are frequent in terms of research focus, because offspring fronds emerge from them and remain connected. Considering fronds of the great duckweed as leaf-like structures that float on the surface of water, the four-frond colony of *S. polyrhiza* represents three generations of mother, daughter, and granddaughter plants of different ages. Therefore, although a relatively minimal tissue differentiation exists among them, we presume that it must influence differently on the response of individual plants to Cd-induced toxicity. This study's objective was to determine if there are age-linked changes in the photosynthetic efficiency of *S. polyrhiza* fronds exposed to different Cd concentrations. If age is a significant factor, how does it influence Cd toxicity dose response, and how and why do the reaction to Cd in the photosynthetic apparatus differ between mothers and daughters and between old and young plants? Also, the goal was to determine the time course of Cd's adverse effects on biochemical, physiological, and morphological changes. Furthermore, short-term assessments of adverse effects are essential for populations at higher risk of environmental pollution.

## Materials and Methods

### Plant Material and Experimental Setup

Cultures of the great duckweed, *Spirodela polyrhiza* (L.) Schleiden (RDSC Clone ID 5634), originally sourced from a drainage canal in Nature Park Kopački Rit, Croatia were grown in a modified Steinberg nutrient solution (Steinberg, 1946) under controlled aseptic conditions in 3-L culture flasks. The nutrient solution was changed every 7 to 10 days and was composed of 3.46 mM KNO_3_, 1.25 mM Ca(NO_3_)_2_, 0.66 mM KH_2_PO_4_, 0.072 mM K_2_HPO_4_, 0.41 mM MgSO_4_, 1.94 μM H_3_BO_3_, 0.63 μM ZnSO_4_, 0.18 μM Na_2_MoO_4_, 0.91 μM MnCl_2_, 2.81 μM Fe(III)EDTA, 1 and 0.22 EDTA; pH was 5.5 ± 0.1(ISO, 2017). The photoperiod in a plant growth chamber was set to 16-/8-h light/dark, and light intensity was 120 μmol·s^−1^·m^−2^ (TLD and CWL 36W; Philips). Temperature was set to 21/25 °C in the dark/light.

For pre-cultivation, cultures were started with a single two-frond colony (mother and daughter fronds) in 6-well cell culture plates (Guangzhou Jet Bio-Filtration Co., Ltd., China), with one colony per well. After 72 h of pre-cultivation, colonies with four fully developed fronds were inoculated into 10 ml of a nutrient solution with different cadmium concentrations on a new set of 6-well culture plates. Pre-cultivation and experiment conditions were the same as cultivation conditions regarding photoperiod, light intensity, and temperature. Growth conditions during pre-cultivation enabled an approximately doubling time of 2.5 days as required by standard protocols (ISO, 2005, 2017). Also, growth rates of *S. polyrhiza* stock cultures were monitored 14 days before the start of the experiment to check the validity and meet the conditions of the standard protocols. The cadmium chloride anhydride (≥99 %; Fluka Chemie, Switzerland) exposure series applied in a modified Steinberg (StMo) medium included the following nominal concentrations of Cd: 0 (C0, control), 7 (C1), 14 (C2), 28 (C3), 56 (C4), 111 (C5), 222 (C6), 445 (C7), and 890 μM (C8). The chosen concentration range allowed for measurements of stronger immediate and short-term effects and enabled better differentiation among the fronds. Since the great duckweed develops offspring vegetatively from meristematic pockets at the frond's proximal end, the maternal frond is the oldest and is designated as MF, mother frond; two new offspring develop from it: DF_1_, the first daughter frond and DF_2_, the second daughter frond. The granddaughter frond (GDF) originates from the first daughter frond. All measurements were made on individual fronds, separated before measurement gently by hand, to determine Cd's effect on differently aged and developed plants. Also, measurements were made after 6, 24, and 72 h of exposure to different Cd concentrations.

### Pigment Content

Carotenoids (Car *x*+*c*), chlorophyll *a* (Chl_*a*_), and chlorophyll *b* (Chl_*b*_) of different *S. polyrhiza* fronds were determined spectrophotometrically from 0.1 g of fresh weight samples in three replicates. Plant samples were extracted with pure acetone containing magnesium hydroxycarbonate (0.5% w/v). The extract was centrifuged (18,000 g, 4°C, and 15 min), and after several re-extractions, absorbances were recorded at 470, 644.8, and 661.6 nm (Analytik Jena, Specord 40). Pigment contents were calculated according to Lichtenthaler and Buschmann (2001):


$$\begin{array}{l} Chl_{a}\left(\frac{\mu g}{ml}\right)=11.24A_{661.6}-2.04A_{644.8}\\ Chl_{b}\left(\frac{\mu g}{ml}\right)=20.13A_{644.8}-4.19A_{661.6}\\ Car_{\left(x+c\right)}\left(\frac{\mu g}{ml}\right)=\left(1000A_{470}-1.9\;Chl_{a}-63.14Chl_{b}\right)/214 \end{array}$$

### Chlorophyll Fluorescence Measurements

Chlorophyll fluorescence was measured in every frond, in the four-frond colony, control, and treatment groups after 6, 24, and 72 h of exposure. Each measurement was performed on six replicates with a Handy PEA^+^continuous-excitation type chlorophyll fluorescence analyzer (Hansatech, United Kingdom). The plants were previously adapted to the dark for 30 min. The transient was induced with a red-light pulse of 3,000 μmol·m^−2^·s^−1^ and analyzed using the JIP-test (Strasser and Strasser, 1995; Stirbet et al., 2014; Tsimilli-Michael, 2020). For a detailed evaluation of the OK, OJ, JI, and IP phases, transient curves were normalized as relative variable fluorescence at time t as follows: $V_{t}=\frac{\left(F_{t}-F_{0}\right)}{\left(F_{m}-F_{0}\right)}=\Phi_{F}\left(t\right)$, where Φ_*F*_(*t*) is the fluorescence yield. Kinetic differences were calculated from the relative variable fluorescence by subtracting the transient of stressed and control plants. For detailed definitions and explanations of the JIP test parameters, refer to Supplementary Table 1 (Stirbet and Govindjee, 2011; Kalaji et al., 2016, 2017; Stirbet et al., 2018; Tsimilli-Michael, 2020).

### Oxygen Exchange Measurements

Oxygen exchange rate was measured with a Clark-type oxygen electrode and a Chlorolab2^+^ system (Hansatech, United Kingdom) at 21°C. Free-floating species, like *S. polyrhiza*, take up most of the carbon needed *via* gaseous CO_2_ from the air. Under laboratory conditions, in a carbonate-free medium, 63% of the carbon used by these plants in photosynthesis comes from the atmosphere. However, duckweeds take 86% carbon from aqueous inorganic carbon (Filbin and Hough, 1985; Landolt and Kandeler, 1987). Therefore, to estimate O_2_ exchange rates accurately, we used a 0.1 M bicarbonate buffer (15 ml 0.1 M K_2_CO_3_/85 ml 0.1 M NaHCO_3_, adjusted to pH 8.9) that provided a constant saturating CO_2_ concentration in which *S. polyrhiza* fronds were placed (Walker, 1987). Under continuous stirring, changes in oxygen concentration in the dark and light (at 120 μmol·m^−2^·s^−1^ of photosynthetically active radiation) were recorded. Net O_2_ exchange rates were calculated per phase by determining the slope of O_2_ exchange curves over 5 min of dark and light periods in three consecutive repetitions (30 min). The net result of oxygen evolution was observed as the sum of all oxygen fluxes between the buffer medium and *S. polyrhiza* fronds (here, the buffer was the substrate for gas exchange). These fluxes included gross O_2_ production (the rate of water splitting at PSII), the oxygenation of Rubisco, mitochondrial respiration, photorespiration (if present), and all other metabolic processes (e.g., the Mehler reaction and nitrate reduction). Oxygen uptake rate during the dark was an estimate of mitochondrial respiration (dark respiration). Both oxygen evolution and uptake rate represent metabolic rates. All the measurements were replicated three times, and rates were normalized to plant sample fresh weight (μmole O_2_ g^−1^FW·h^−1^).

### Light and Transmission Electron Microscopy

Anatomical observations were performed on mother and daughter plants exposed to C1 (7 μM Cd) and C3 (28 μM Cd) concentrations after 6, 24, and 72 h of exposure with a transmission electron microscope (TEM) and after 72 h with a light microscope. For light microscopy, cutoffs of *S. polyrhiza* fronds were fixated (24 h, 4°C) with 1 % glutaraldehyde in a 50-mM phosphate buffer (pH = 6.8). Gradual dehydration was carried out at room temperature in 1-h steps with 2-methoxyethanol, 96 % ethanol, n-propanol, and n-butanol. Pre-infiltration was performed for 24 h with equal parts of an infiltration solution and n-butanol. Dehydrated specimens were embedded into a Leica Historesin Embedding kit. Semi-thin (3 μm) sections were stained with a Lugol solution to detect differences in starch distribution and observed with a light microscope (Carl Zeiss 106; Jena, Germany) and Moticam 350.

For TEM, a primary fixation of *S. polyrhiza* frond tiny cutoffs was performed for 60 min with 1% glutaraldehyde in a 50-mM cacodylate buffer (pH 7.2) while gently evacuating to remove air in the tissue. After rinsing with a cacodylate buffer for 3 × 10 min, post-fixation was carried out in 1% OsO_4_ dissolved in a 50-mM cacodylate buffer (8–12 h, in the dark at 4°C). Gradual dehydration was performed by passing the samples through increasing ethanol concentrations and propylene oxide at the end. Dehydrated specimens were embedded into Spurr's propylene oxide and epoxy resin mixture in three 2–3 h infiltration steps (2:1, 1:1, 1:2) while gently agitating vials at room temperature. The specimens were then placed into fresh Spurr's mixture for 4–5 h at 40°C, replaced with a pure new resin, and, finally, 3–4 h later, allowed to polymerize at 60–65°C for 48 h. Ultra-thin (80 nm) sections were cut with a Leica EM UC7 ultramicrotome (Leica Microsystems, Vienna, Austria) and post-stained with 0.02 M lead citrate dissolved in fresh distilled water containing 0.16 M sodium hydroxide (3 min) and 2% uranyl acetate dissolved in distilled water (10 min). The sections were examined in Zeiss Libra 120 Plus TEM (Carl Zeiss AG, Oberkochen, Germany). Micrographs were taken with an XF416 4k camera (Tietz Video and Image Processing Systems GmbH, Gauting, Germany).

### Statistical Analysis

To determine how *S. polyrhiza* pigment content responds to Cd treatment during 72 h of exposure and the amount of variation in pigment content accounted for by frond age, we conducted a two-way analysis of covariance (ANCOVA) with frond age as a controlling variable (covariate). When necessary, variables were log-transformed to meet the statistical assumptions of the ANCOVA. The mean difference between treatment and control was calculated for each tested parameter to assess similarities in response to Cd between different generations of fronds. As the calculation of the mean difference does not consider the standard deviation within the groups, Hedges effect size was calculated. Effect size is a relevant interpretation of an estimated magnitude of an effect from effect statistics or, in other words, a quantitative measure based on overall standard deviation (both control and treatment). The bigger the effect size, the higher the increase/decrease in the treatment's specific parameter (Hedges and Olkin, 1985). The statistical significance (acquired by *t*-test and confidence interval statistics) of the differences between treatment and control in individual parameters coincides with Cd treatment's high to very high effect. By combining the estimation of effect size and confidence interval statistics, we obtained more information than *p*-value testing alone provides (Nakagawa and Cuthill, 2007). Presentation and interpretation of effect size reduced the prevailing misinterpretation of null-hypothesis and *p*-value testing and improved scientific results, providing a meaningful evaluation of treatment effect and its comparison among fronds and exposure times.

Furthermore, principal component analysis (PCA), a multivariate statistical technique, was conducted to reduce a large set of Chl fluorescence parameters to the most informative ones (Goltsev et al., 2012; Kalaji et al., 2017). Also, PCA was conducted to investigate a combined effect of cadmium concentrations and frond age on the structure of variability among fronds and correlations between Chl fluorescence parameters with pigment contents. Factor loadings on the first two principal components (PCs) were shown after oblique (direct oblimin) rotation. At the same time, a hierarchical k-means clustering algorithm on main features was used to obtain optimal cluster solutions (Bussotti et al., 2020). Data presentations were made in Excel and statistical analyses were conducted with Statistica 13.5 (© 1984–2018 TIBCO Software Inc.).

## Results and Discussion

### Pigment Content

A four-frond colony of the great duckweed *S. polyrhiza* represents generations of different plant ages: mother, daughter, and granddaughter plants. Plant pigment content changes with age, so if we control the age response, we will improve the likelihood of finding a significant two-way interaction if one exists. Therefore, the results of the two-way ANCOVA model explained 74 to 83% of the variability in pigment content and their ratios (Table 1). Cd concentrations, exposure time, and their significant interaction indicated that the response rate differs between treatments, thus bringing important information explaining the variability of chlorophyll concentrations. The duration of Cd exposure accounted for the most variation in pigment content as the most influential variable; as shown in Table 1, the Fisher F-statistic and effect size are highest for the time variable. A more detailed overview of the statistical analysis results is given in Table 2.

**Table 1:** Two-way analysis of covariance (ANCOVA) of pigment concentrations and their ratios measured in *Spirodela polyrhiza* fronds of different age (FA: MF, DF_1_, DF_2_, and GDF) exposed for 6, 24 and 72 h (time) to different concentrations of cadmium (Cd: 0–890 μM).

	**df**	**Chl *a***	**Chl *b***	**Chl (*a*+*b*)**	**Car (*x*+*c*)**	**Chl *a*/*b***	**Chl/Car**	**η^2^**
R^2^		0.83	0.74	0.81	0.83	0.53	0.84	
Cd	8	38.8***	17.1***	33.1***	54.9***	13.2***	85.8***	0.512
Time	2	429.0***	272.8***	397.8***	292.1***	95.3***	83.8***	0.572
FA	1	2.2^ns^	0.02^ns^	1.3^ns^	9.8**	0.5^ns^	25.1***	0.286
Cd × Time	16	8.8***	6.1***	8.3***	12.5***	3.3***	9.6***	0.306
Cd ×	16	1.6^ns^	1.0^ns^	1.5^ns^	2.3**	1.0^ns^	1.3^ns^	0.088
Time × FA	
Error	292							

df, Degrees of freedom; F, Fisher's F-test statistic; R^2^, adjusted whole model regression coefficient; η^2^ partial eta-squared (effect size). Significant levels were: ^ns^p > 0.05; **p < 0.01; ***p < 0.001.

**Table 2:** Analysis of the differences between categories (Cd × time) with FA as a covariate.

**Exposure time**	**Cd mM**	**Chl *a***	**Chl *b***	**Car(*x*+*c*)**
		**(μg g^**−1**^ FW)**	**(μg g^**−1**^ FW)**	**(μg g^**−1**^ FW)**
6 h	0	538.2 ± 26.2^defghi^	187.3 ± 10.6^de^	148.9 ± 5.5^defg^
	7	503.5 ± 22.9^def^	177.8 ± 7.5^d^	147.1 ± 4.8^cdef^
	14	495.3 ± 24.3^def^	178.2 ± 8.6^d^	147.7 ± 5.6^cdefg^
	28	494.6 ± 18.7^de^	172.5 ± 6.1^cd^	150 ± 3.3^defg^
	56	506.4 ± 22.2^def^	180.2 ± 7.6^d^	153 ± 3.4^defghi^
	111	509.4 ± 15.4^def^	179.2 ± 4.7^d^	151.1 ± 2.7^defgh^
	222	498.5 ± 14.4^def^	176.7 ± 6.1^cd^	146.1 ± 2.5^cdef^
	445	535.3 ± 12.6^defgh^	188.6 ± 5.9^def^	150.5 ± 3.3^defgh^
	890	537.3 ± 8.1^defghi^	185.6 ± 2.4^de^	149.3 ± 2.1^defg^
24 h	0	565.4 ± 9.1^defghi^	188 ± 4.7^def^	151.2 ± 2.8^defgh^
	7	594.4 ± 13.8^efghi^	204.1 ± 5.7^def^	163.1 ± 4.1^defghi^
	14	639.8 ± 9.5^i^	222.5 ± 5.7^ef^	173 ± 2.8^ghi^
	28	629 ± 7.3^hi^	227.4 ± 4.8^f^	176 ± 2.2^hi^
	56	598.8 ± 18^fghi^	224.8 ± 8.2^ef^	171.1 ± 4.8^fghi^
	111	596.4 ± 8^efghi^	221.5 ± 4.4^ef^	178.1 ± 2.5^i^
	222	553.3 ± 13.4^defghi^	199.5 ± 12.3^def^	167.8 ± 4.2^efghi^
	445	486.4 ± 24.2^d^	194.6 ± 7.9^def^	142.4 ± 5.6^cde^
	890	506.3 ± 19.6^def^	193.6 ± 7.9^def^	140.8 ± 5.9^cd^
72 h	0	622.9 ± 11.1^ghi^	208.1 ± 3.9^def^	168.5 ± 3^fghi^
	7	523.4 ± 27^defg^	195.2 ± 8.5^def^	161.2 ± 8^defghi^
	14	474 ± 28.9^d^	175.1 ± 7.7^cd^	164.3 ± 5.2^defghi^
	28	341.7 ± 17.5^c^	137.3 ± 6.1^bc^	149.8 ± 5.3^defg^
	56	254.4 ± 26.3^bc^	104.8 ± 8.7^ab^	122.8 ± 9.1^c^
	111	171.7 ± 18.5^ab^	80.3 ± 6.2^a^	87.4 ± 6.6^b^
	222	138.6 ± 16.6^a^	69.3 ± 7.1^a^	57.6 ± 5.1^a^
	445	164.4 ± 24.7^ab^	81 ± 10.2^a^	46 ± 5.1^a^
	890	197.2 ± 22.8^ab^	96.6 ± 8.8^a^	40.8 ± 3.1^a^

*The results of pigment content are presented as the mean and standard error of the mean. Values followed by common letters are not significantly different (Tukey HSD test) at the 5% significance level*.

**Figure 1:**
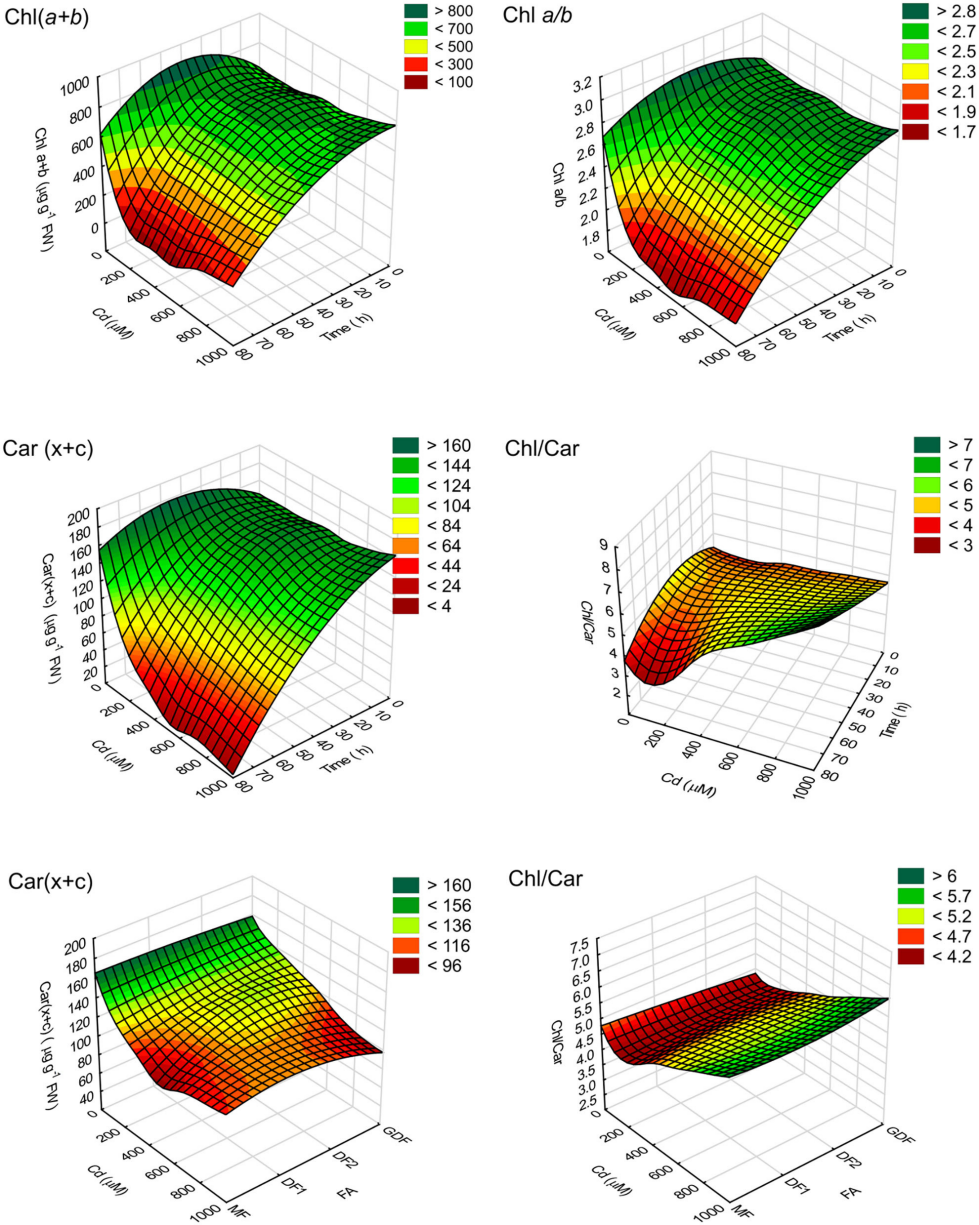
3D response surface plot (distance weighted least squares) of pigment content (μg g^−1^ FW) and their ratios. Plots represent the relationship between time of exposure to different cadmium (Cd) concentrations (C0–C8) reconstructed using an MLP algorithm (4.MLP 6-4-4) of measured chlorophyll concentrations Chl(*a*+*b*), total carotenoids, Car(*x*+*c*), and pigment ratio (Chl *a/b* and Chl/Car).

Since time controls the response of Chl content to cadmium treatments and not the frond age (Table 1), based on pigment content and statistical analysis of measured values, a multilayer perceptron model (MLP) algorithm with a three-layer structure illustrated *S. polyrhiza* response. The surface response 3D plot for the relationship between time and concentration-dependent pigment content in Figure 1 shows that high concentration and longer exposure time resulted in highest reduction in pigment content. The inclined profile of surface plots implies the interacting effect of both factors. Since the ANCOVA showed that fronds do not account for significant variability in chlorophyll content, frond age was used as an input variable with summation in Chl and activation function in Car. The observed decrease in pigment content could be related to either reduction in the rate of chlorophyll biosynthesis by Cd inhibition of protochlorophyllide reductase or chlorophyll degradation (Ci et al., 2010; Song et al., 2019; Franić et al., 2020; Janeeshma et al., 2021). However, carotenoid contents behaved slightly differently in fronds of different ages. Their reduction rate depended on exposure time, Cd concentration, and frond age (Table 1 and Figure 1). Therefore, a response surface plot in Figure 1 shows the relationship between Car content and the ratio Chl/Car with *S. polyrhiza* frond age. The oldest MFs exhibited a more significant and faster reduction of total carotenoids as Cd concentrations were increased. There was also a subsequent increase in total carotenoids with increase in cadmium concentration, most likely due to pigment degradation and accelerated senescence caused by Cd (Krupa and Moniak, 1998; Tkalec et al., 2008).

### Chlorophyll Fluorescence

**Figure 2:**
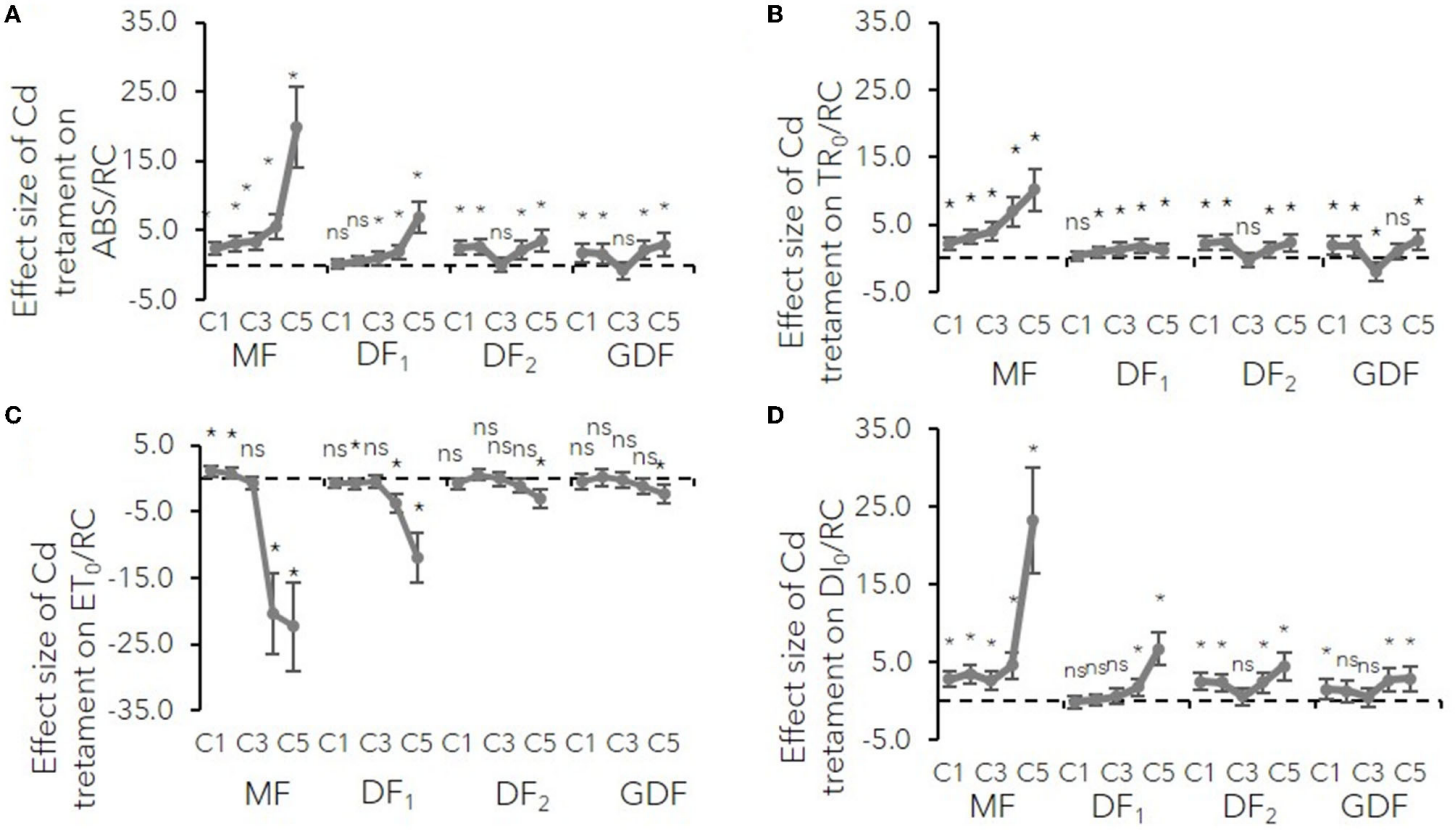
Mean effect sizes (Hedges d) and bias-corrected bootstrap 95% confidence interval for the overall effect of Cd treatment on specific energy fluxes (per reaction center, RC) for **(A)** absorption (ABS/RC), **(B)** trapping (TR_0_/RC), **(C)** electron transport (ET_0_/RC) and **(D)** dissipation (DI_0_/RC). Effect sizes are based on mean differences between treatment and the control (C0) after 72 h of exposure to Cd concentration from 7 μM–C1 to 111 μM–C5. The dashed line shows Hedges d = 0. When confidence intervals overlap zero, the effect sizes are not significantly different from zero (*p*-values for mean differences are obtained by 2-tailed *t*-test, ns for *p* > 0.05; *p < 0.01).

**Figure 3:**
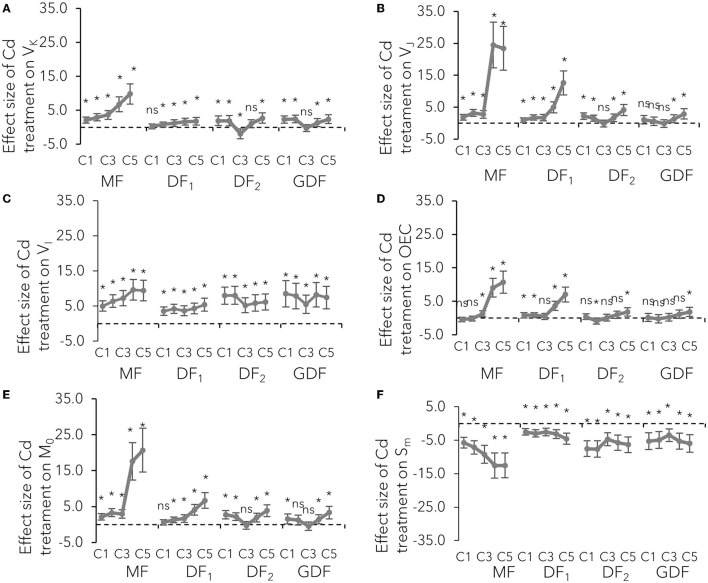
Mean effect sizes (Hedges d) and bias-corrected bootstrap 95% confidence interval for the overall effect of Cd treatment on technical fluorescence parameters: **(A)** relative fluorescence at K step (300 ms–V_K_), **(B)** at J step (2 ms–V_J_), **(C)** at I step (30 ms–V_I_), **(D)** the fraction of O_2_ evolving centers, **(E)** the initial slope that indicates the net closing rate of the reaction centers M_0_ [M_0_ = 4 (F_300μ*s*_-F_0_)/(F_m_-F_0_)], and **(F)** a proxy of electron carriers number per electron transport chain (Sm). Effect sizes are based on mean differences between treatment and the control (C_0_) after 72 h of exposure to Cd concentration from 7 μM–C1 to 111 μM–C5. The dashed line shows Hedges d = 0. When confidence intervals overlap zero, the effect sizes are not significantly different from zero (*p*-values for mean differences are obtained by 2-tailed *t*-test, ns for *p* > 0.05; *p < 0.01).

**Figure 4:**
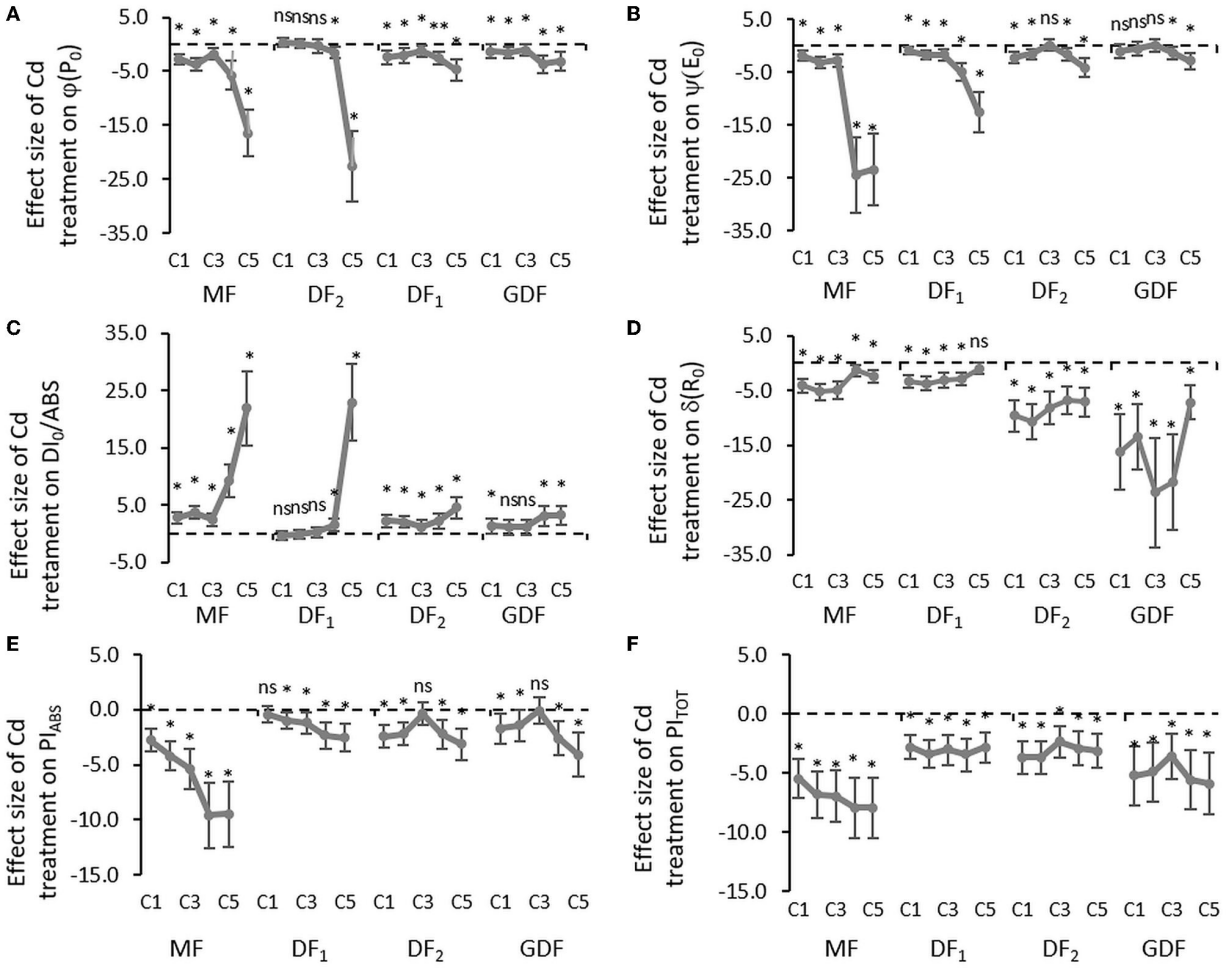
Mean effect sizes (Hedges d) and bias-corrected bootstrap 95% confidence interval for the overall effect of Cd treatment on efficiencies and quantum yield: **(A)** the maximum quantum yield of primary PSII photochemistry that leads to QA reduction (ϕP_0_), **(B)** the probability that a trapped exciton moves an electron into the electron transport chain beyond Q_A_ (ψE_0_), **(C)** quantum yield of energy dissipation in PSII antenna (DI_0_/ABS), **(D)** the efficiency that contains the rate constants for intersystem electron transport and reduction of the end-electron-acceptor (δR_0_), **(E)** performance index (potential) for energy conservation from exciton to the reduction of intersystem electron acceptors, and **(F)** performance index (potential) for energy conservation from exciton to the reduction of PSI end acceptors. Effect sizes are based on mean differences between treatment and the control (C0) after 72 h of exposure to Cd concentration from 7 μM–C1 to 111 μM–C5. The dashed line shows Hedges d = 0. When confidence intervals overlap zero, the effect sizes are not significantly different from zero (*p*-value for mean difference is obtained by 2-tailed *t*-test, ns for *p* > 0.05; *p < 0.01).

The polyphasic rise of Chl fluorescence induction kinetics in *S. polyrhiza* exposed for 6, 24, and 72 h to Cd concentrations ranging from 0 to 890 μM was monitored to localize the action sites of Cd stress in the photosynthetic electron transport chain. For simplification, only the results obtained after 72 h of Cd exposure were selected for graphical presentation in Supplementary Figure 2. However, to reveal the time sequence of events, differential curves of Chl fluorescence transient normalized between O-K, O-J, and I-P steps were shown after 6, 24, and 72 h of Cd exposure (Supplementary Figures 3–5). The effect of cadmium stress on electron transport at the donor and acceptor sides of PSII and PSI was determined from quantitative data of JIP-test parameters. The mean differences of these parameters between treatments and the control are shown in Figures 2–**4**.

The results show how cadmium affects different photosynthetic electron transport segments relative to their structure and function. Connectivity between reaction centers and their antenna complexes was expressed as an L-band of Chl fluorescence transient normalized between O-K steps (Stirbet and Govindjee, 2011). The L-band's positive deviations increased their amplitude with Cd concentration, exposure time, and frond age (Supplementary Figure 3). A higher amplitude of L-band in mother plants meant a more significant loss of antenna connectivity due to Cd stress. Moreover, the decrease in the number of active reaction centers within the chlorophyll bed by increasing cadmium concentrations was confirmed by a more significant increase in antenna size estimation or ABS/RC in older mature plants (MF and DF_1_, Figure 2A). Younger plants had a similar rise in ABS/RC (from 10 to 20 %) regardless of how much Cd they were exposed to (C1–C5). This increase meant either active reaction centers were inactivated or the apparent antenna size increased. Either way, in low concentrations and younger fronds, a slight rise in ABS/RC was probably a protective mechanism from photooxidative damage and excess in absorbed light energy (Yusuf et al., 2010).

In the MFs, an increase in ABS/RC was accompanied by increased trapped photon flow (TR_0_/RC, V_K_) with the same trend. An increase in TR_0_/RC was accompanied by a decrease in the maximum quantum yield of PSII (ϕP_0_), indicating that the probability of a trapped photon ending up in the RC and causing a photochemical event decreases with increase in Cd concentration and with greater effect size in the MFs than in the DFs. This increase in trapping and reduction of maximum quantum yield at higher concentrations was probably due to the rise in the number of inactive centers, which dissipated the total energy as heat, not fluorescence, as confirmed by the ratio of dissipation energy to the number of active RCs (Figures 2, **4**). In the younger plants (DF and GDF), the mentioned minor increase in ABS/RC was accompanied by a similar rise in TR_0_/RC. Simultaneously, the maximum quantum yield of PSII photochemistry underwent minor changes indicating that antenna size supplied excitation energy to active RCs, and that there was a probable increase in the young plants' functional antenna size (Figures 2–**4**).

The donor side of PSII is a place of the catalytic site of water cleavage and formation of the dioxygen molecule (Stirbet and Govindjee, 2011). Differential curves of Chl fluorescence transient, calculated between O-J steps representing K-band (Supplementary Figure 4), variable fluorescence at K step, and the fraction of O_2_ evolving centers (OEC), were used to examine the PSII donor side's condition (Figure 3). K-bands were strongly expressed after 72 h of Cd exposure. The amplitude increased with increase in Cd concentration (Supplementary Figure 4), while the size of the effect increased with plant age (Figures 3A,D). This V_K_ increase on the normalized O-J curve signified that OEC activity was damaged, and that damage was more intense in the older fronds.

The maximal rate of closed reaction center fraction (M_0_) accumulation, the probability by which the captured exciton moves the electron from the primary acceptor Q_A_ to Q_B_ (ψE_0_) described by variable fluorescence at J step (V_J_), and the quantum yield of electron transport from Q${}_{\text{A}}^{-}$ to PQ PSII (ϕE_0_) were used to examine the effect of Cd on the PSII acceptor side. The initial slope of the relative variable fluorescence curve (M_0_) indicates that the net closing rate of the reaction centers was increased by about 200% in MFs exposed to 56 (C4) and 111 (C5) μM Cd after 72 h, as opposed to the about 37 and 26 % increase in younger fronds (DF_2_, GDF) exposed to the same concentrations of Cd (Figure 3E). However, some slight differences in M_0_ increase among the fronds (range was from 16 to 18% increase) were noted when exposed to the lowest Cd concentration. At the same time, the probability that a trapped exciton moves an electron into the electron transport chain beyond Q_A_ (ψE_0_) was decreased from 20% in young plants up to 80% in mother plants when exposed to C4 and C5 Cd after 72 h (Figure 4B). This decrease was concentration-dependent in mother fronds, but this concentration gradient was not observed in younger fronds. Therefore, the electron transport on the acceptor side of PSII between Q_A_ and Q_B_ was inhibited by Cd concentrations above 28 (C3) μM. Cadmium effect increased with plant age and exposure time (Supplementary Figure 4, Figures 3, 4).

Considerable decline in the probability that an electron is transferred from the reduced intersystem electron acceptors to PSI end-electron acceptors (δR_0_) indicated that intersystem electron transport was inhibited, and that the size of the effect was more extensive in younger fronds than mother fronds (Figure 4D). However, cadmium increased the number of non-reducing reaction centers (V_I_) with almost an equal effect size (Figure 3C). Electron transfer efficiency from PSII to PSI end-electron acceptors (ΔV_IP_) was reduced by 20 % in all the fronds and concentrations within the first 6 h of exposure (Supplementary Figure 5). After 24 h, a concentration gradient was formed in older fronds, while same as with all the other parameters, no gradient was found in younger fronds. With more prolonged exposure to Cd, electron transfer efficiency from PSII to PSI end-electron acceptors was inhibited by 20–50% in the MFs, around 50 % in the DF_1_s, and 40–60% in the younger fronds (DF_2_s and GDFs). This lower effect of higher cadmium concentrations on older fronds resulted from more significant inhibition in previous electron transport steps in these fronds. In fronds exposed to lower Cd concentrations, there was an increase in the reduction rate of PSI end-electron acceptors, followed by a subsequent decrease. This traffic jam caused by a blockage on the acceptor side slowed down the electron transfer through both photosystems, causing a reduction in PI_ABS_ and PI_TOT_ (Figures 4E,F).

#### PCA and Clustering

**Figure 5:**
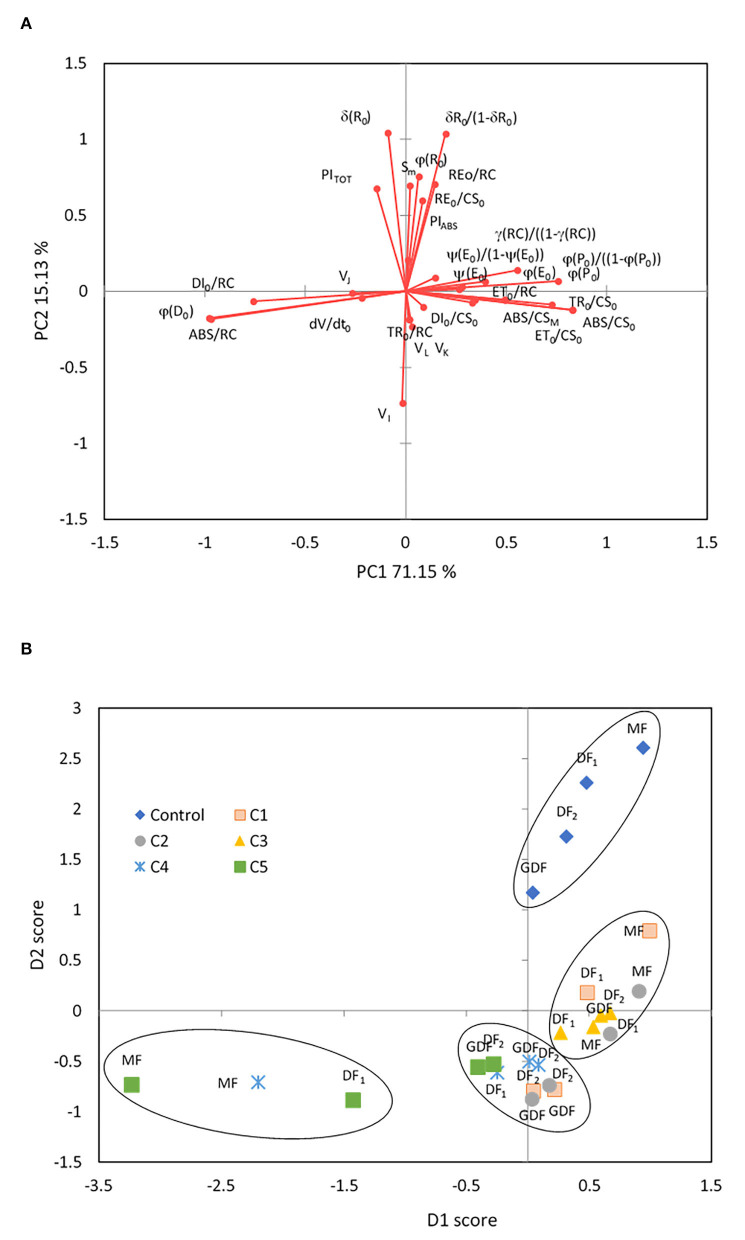
Principal component analysis of 31 Chl fluorescence parameters in control and response to Cd treatment from 7 (C1) to 111 μM (C5) after 72 h of exposure. Principal component 1 (PC1) accounted for 71.2 % of the variance. Principal component 2 (PC2) accounted for 15.1 % of the variance. **(A)** Factor loadings on the first two principal components (PCs) are shown after oblique (direct oblimin) rotation. **(B)** The two PC projections after oblimin rotation with factor scores are sorted by frond age and Cd concentrations. Plotted points belong to centroids of differently aged plant fronds (MF, mother frond; DF_1_, first daughter frond; DF_2_, second daughter frond; GDF, granddaughter frond).

Furthermore, a PCA was conducted to test the combined effect of Cd concentrations and frond age on the correlation between the selected Chl fluorescence parameters of *S. polyrhiza*. The first two principal components explained the variations of 86.3 % in the total chlorophyll fluorescence variables. Principal component 1 (PC1) accounted for 71.2% of the variance, and principal component 2 (PC2) accounted for 15.1% of the variance (Figure 5A). Positive loadings characterized the first component with 61.9% contribution were ABS/CS_0_, ABS/CS_M_, TR_0_/CS_0_, ET_0_/RC, ET_0_/CS_0_, ϕP_0_, ψE_0_, ϕE_0_, γRC/1-γRC, ϕP_0_/1-ϕP_0_, ψE_0_/1- ψE_0_, Chl_*a*_, Chl_*b*_, Car, and Chl *a*/*b*. PC1 included negative loadings in the opposite position with a 32.5 % contribution of parameters connected to dissipation mechanisms ABS/RC, DI_0_/RC, DI_0_/CS_0_, ϕD_0_, V_J_, and dV/dt_0_. Therefore, similar to the conclusions of Bussotti et al. (2020), PC1 represents the capacity of PSII to capture and retain photons and move electrons into the electron transport chain in the first photochemical phase.

The overall function of PSII as maximum quantum yield (ϕP_0_) was highly correlated with parameters related to the efficiency of the water-splitting complex and the density of active reaction centers on the donor side of PSII (ϕP_0_/1- ϕP_0_, γRC/1-γRC), the quantum yield of photoinduced electron transport at the acceptor side of PSII (ψE_0_, ϕE_0_), and carotenoid content (*r* = 0.82, *p* < 0.01). Simultaneously, strong negative correlations were determined between maximum quantum yield and parameters of dissipation mechanisms and PSII apparent antenna size. PC2 was characterized by positive loadings with 68.3 % contribution related to reduction of end electron acceptors (δR_0_, ϕR_0_, RE_0_/RC, RE_0_/CS_0_, δR_0_/1-δR_0_) that characterize IP-phase, the pool size of electron carriers (S_M_, N), and the total performance on absorption basis (PI_TOT_). The total contribution of negative loadings on the opposite side of PC2 was 24 % and included parameters connected to the disconnection of the tripartite system (RC-core antenna-LHC) described by variable fluorescence at L-band (V_L_, with 5.5 % contribution), inactivation of the oxygen-evolving system described by variable fluorescence at K-band (V_K_, with 4.9 % contribution), the reduction of PSI electron acceptors related to variable fluorescence at I-band (V_I_, with 8.9% contribution), and trapped photon flow (TR_0_/RC, with 4.9% contribution) per active PSII. Accordingly, PC2 represents the thermal phase, which correlates well with the acceptor side of PSI and influences the overall photosynthetic behavior and the whole linear electron transport (Bussotti et al., 2020).

The four groups, superimposed in the chart (Figure 5B), were generated using the hierarchical k-means clustering algorithm on principal components. By analyzing all chlorophyll fluorescence parameters together, a clear separation of the control plants into a distinct group is shown in correspondence with positive values of PC2, meaning that after 72 h, only the control fronds maintained fully functional and undisturbed linear electron transport. In all the other fronds, Cd harmed at least the thermal phase of photosynthesis. Hierarchical k-means clustering separated stressed fronds into three distinctive groups defined by their response to Cd concentrations. The first group was correlated positively with PC1 and consisted of mother plants (MFs, DF_1_s) exposed to low concentrations of Cd (C1-C3) and daughter plants (DF2s, GDFs) exposed only to the C3 Cd concentration. Their photosynthesis was determined by the functionality of primary electron acceptance at the PSII level. On the opposite side along the PC1 axis, the most stressed fronds are the MFs exposed to high Cd concentrations (MFs at C4 and C5, and DF1s at C5). This group was characterized by closed and inactive reaction centers, meaning that all the energy outflow they would generally use for photochemistry dissipated as heat, inhibiting photosynthesis. The third group of stressed plants is located orthogonally to PC1. It correlates with negative loadings of PC2 or the first phases of fluorescence transient, L- and K-bands, although with lower component loadings. This group also correlates with increased non-reducing reaction centers (V_I_) and subsequent thermal phase reduction on the opposite side along the PC2 axis. These plants are mainly young fronds (DF_2s_ and GDFs) exposed to the lowest and highest Cd concentrations, indicating that an increase in V_I_ does not necessarily mean an increase in non-reducing reaction centers but could mean less functional reaction centers. The effect of C4 and C5 on young fronds was also related to dissipative mechanisms (negative loadings of PC1). In contrast, low concentrations most probably caused an increase in functional antenna size (positive loadings of PC1).

**Figure 6:**
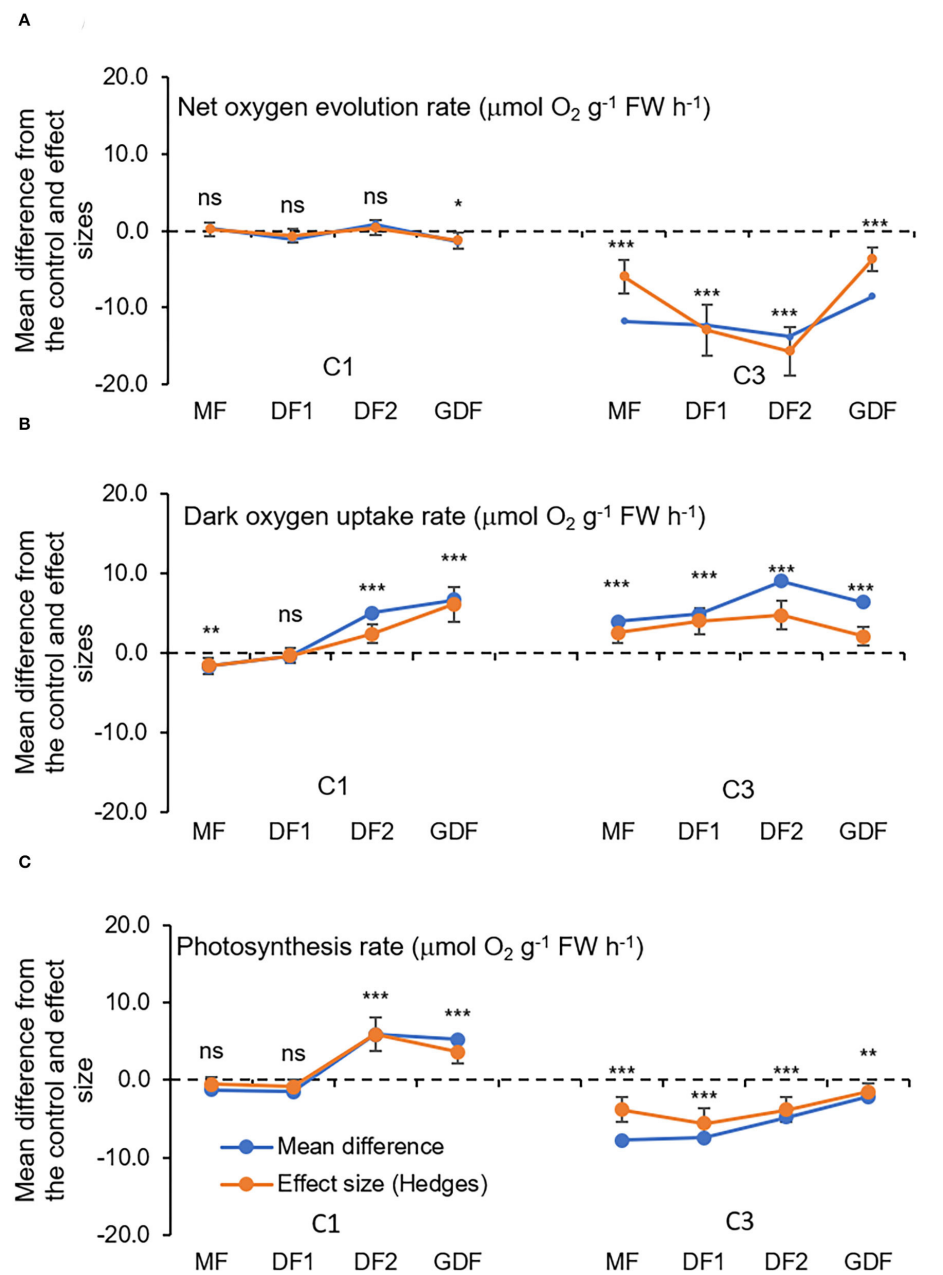
Mean differences between Cd treatment (7μM, C1 and 28 μM, C3) and the control (C0, StMo) and treatment effect sizes (Hedges d) with bias-corrected bootstrap 95% confidence interval for O_2_ exchange rates(μmol O_2_ g^−1^ FW h^−1^): **(A)** net uptake rate during the dark period, **(B)** net oxygen evolution rate during the light period, and **(C)** apparent photosynthesis rate obtained by subtracting uptake rate from net oxygen evolution rate. Each point represents a total of nine replicates. The dashed line shows Hedges d = 0. When confidence intervals overlap zero, the effect sizes are not significantly different from zero (*p*-value for mean difference is obtained by 2-tailed *t*-test, ns for *p < 0.05; **p < 0.01; ***p < 0.001).

**Figure 7:**
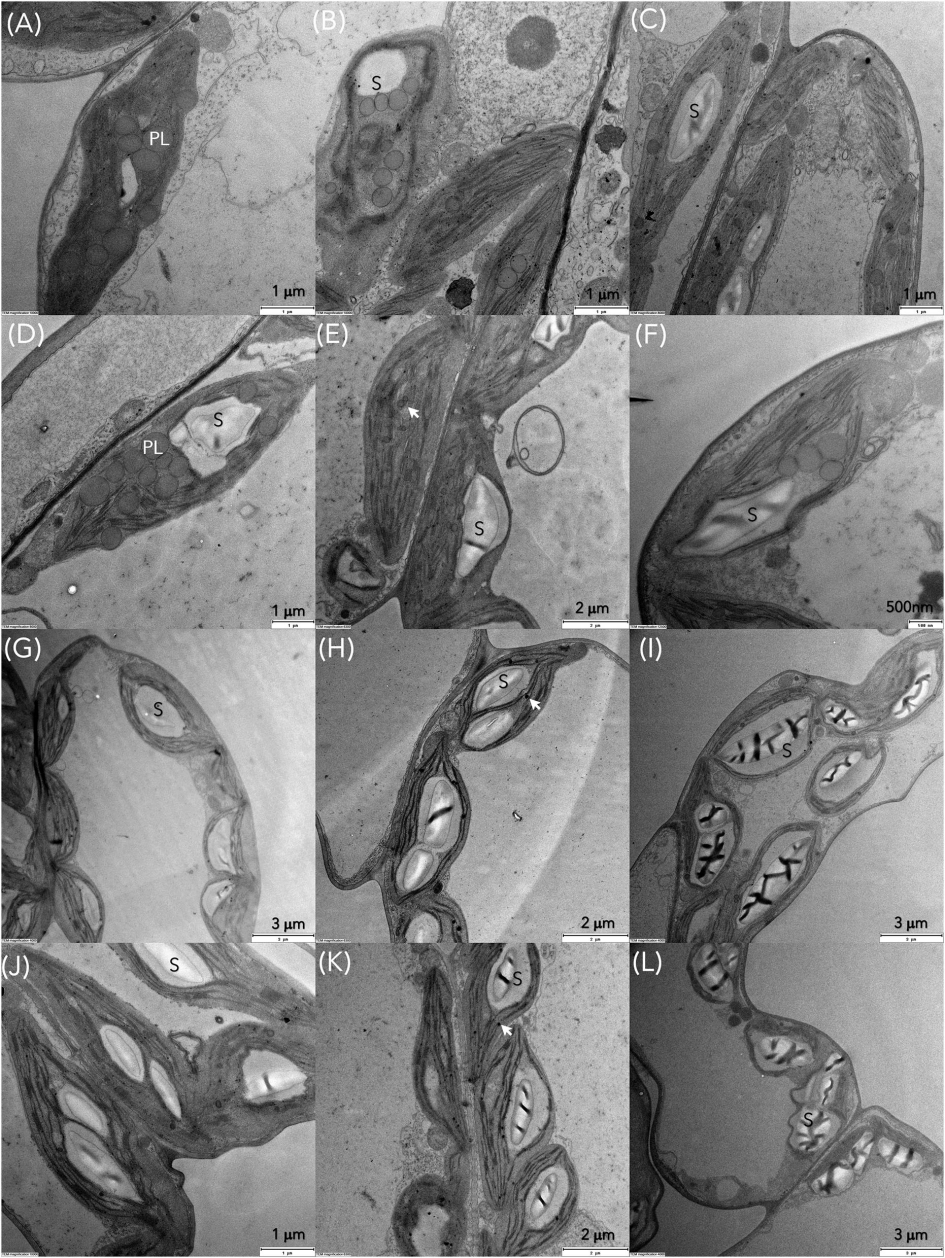
Transmission electron microscopy (TEM) images of *S. polyrhiza* mother fronds (MFs) after exposure for 6, 24, and 72 h to Cd concentrations **(A–C)** C1 and **(D–F)** C3 and granddaughter fronds (GDFs) after 6, 24, and 72 h of exposure to **(G–I)** C1 and **(J–L)** C3 Cd. PL and black arrow represent plastoglobules, S is starch grain.

**Figure 8:**
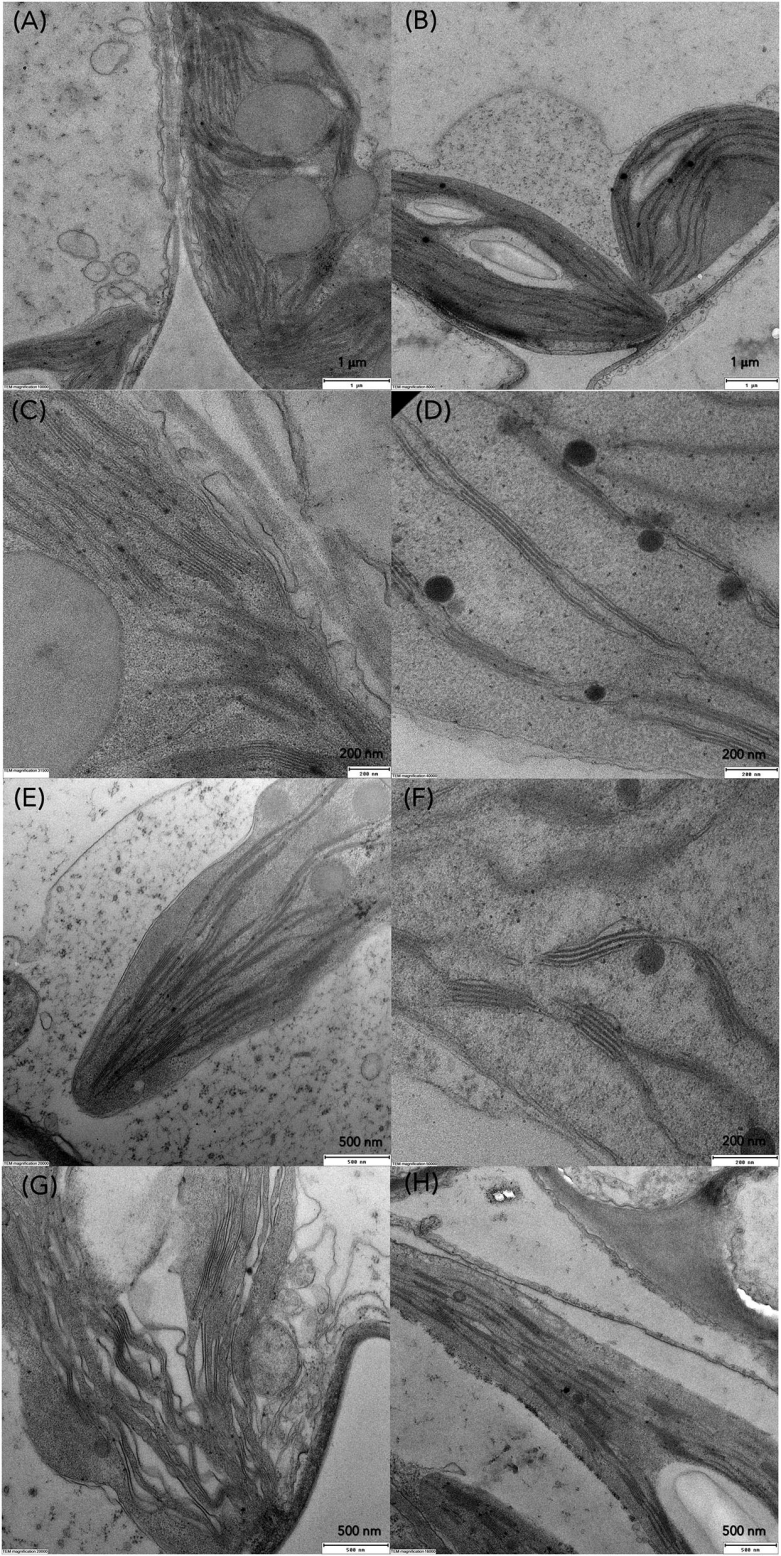
TEM micrographs of chloroplasts in control **(A,C)** MFs, **(B,D)** GDFs, and changes in thylakoid organization in *S. polyrhiza* chloroplasts of MFs and GDFs exposed to **(E,F)** C1 and **(G,H)** C3 Cd concentration after 24 h.

Based on the analysis of chlorophyll fluorescence parameters (Figures 3–**8**) of mother and daughter plants and their distinct response to the C1 and C3 treatments (Figure 5B), further analyses of *S. polyrhiza* fronds were focused on their reaction to these concentrations.

### Oxygen Exchange Measurements and Chloroplast Ultrastructure

The presented results of oxygen exchange rates refer to measurements after exposure for 72 h to C1 (7 μM) and C3 (28 μM) Cd concentrations and represent a mean difference between treatment and control and treatment effect sizes (Figure 6). In mother fronds, the net oxygen uptake during the dark after 72 h of exposure to C1 was significantly decreased by 28 % relative to the control MFs. However, the net oxygen evolution rate was increased, although not significantly (6%). In the youngest GDF fronds, and the dark oxygen uptake rate after 72 h of exposure to C1 significantly increased relative to the control GDF fronds by 114 %. At the same time, the net exchange of O_2_ was significantly reduced by 53%. The oxygen uptake rate in fronds exposed to C3 increased considerably in all the fronds, from 75% in the oldest MFs to 124% in the younger DF_2_s. On the other hand, the net O_2_ evolution rate was significantly decreased from 183 % in MFs up to more than 300% in more immature fronds (DF_2_s and GDFs), a six-fold decrease in contrast to C1.

Prasad et al. (2001) determined that *Lemna trisulca* exposed to low concentrations of Cd increases its respiration rates, mobilizing metabolism, and that under severe stress, respiration decreases, indicating metabolism damage. According to Amthor (2000), adverse growth conditions will increase respiration relative to photosynthesis and, thus, reduce the total carbon yield in the plant. Under stressful conditions, significantly increased respiration rates of *S. polyrhiza* indicated an improvement in physiological activities, whether the rate of respiration or photosynthesis. Improving photosynthetic activities may subsequently increase growth rates (Jager et al., 2013). However, this hormetic effect of Cd on respiration and photosynthesis may be an adaptive response of the plant organism to distribute the amount of energy available to suppress losses. Because low concentrations can cause partially repairable tissue injuries, the metabolic rate will increase by activating mechanisms involved in mitigating the harmful effects of Cd. Why? To repair stress-induced damage by, e.g., removing reactive oxygen species (Tkalec et al., 2008) or binding Cd to starch (Higuchi et al., 2015). Repair is a process that consumes energy and increases respiration rate (Jager et al., 2013). As the respiration rate increases, substrates are diverted from growth. Consequently, the growth rate of *S. polyrhiza* decreases, even at the lowest cadmium concentration (Supplementary Figure 1).

Transmission electron microscopy (TEM) was conducted to compare ultrastructural changes in *S. polyrhiza* chloroplasts upon exposure for 6, 24 and 72 h to C1 (7 μM) and C3 (28 μM) Cd concentrations in older and younger fronds (Figure 7). In the control older fronds, the observed chloroplasts with well-developed granum stacks connected by intergranal thylakoids, small starch grains (rarely), and several large plastoglobuli (Figures 8A,C) are consistent with their age. In the younger control fronds (GDFs), elongated intergranal thylakoids in one or two layers are embedded in the chloroplast stroma with rarely formed granum stacks (Figures 8B,D), small plastoglobuli, and regular shaped starch grain (one or two). Single or paired thylakoids without large granum stacks could be associated with a reduced risk of photoinhibition under stress (Krupinska et al., 2012). Cd-induced changes in the ultrastructure of the chloroplasts were visible even after 6 h of exposure to the lowest concentration used.

**Figure 9:**
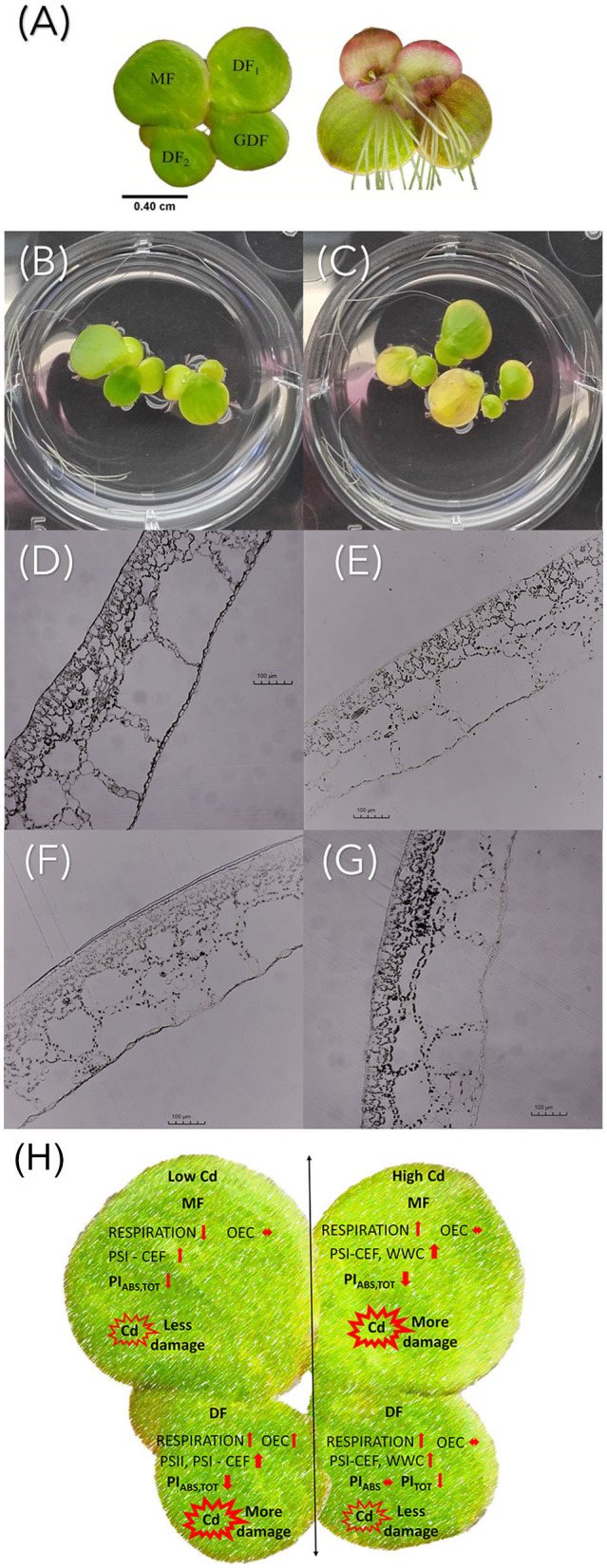
**(A)** *S. polyrhiza* four-frond colony. MF, mother frond; DF_1_ and DF_2_, first and second daughter fronds; GDF, granddaughter frond. Visual symptoms of Cd treatment with **(B)** C1 and **(C)** C3 after 72 h of exposure. Light microscopy images of *S. polyrhiza* **(D,E)** mother and **(F,G)** granddaughter frond cross-sections treated with diluted Lugol solution after exposure to **(D,F)** C1 and **(E,G)** C3 Cd for 72 h. The scale bar is 100 μm. **(H)** Graphical summary of cadmium-induced changes in *Spirodela polyrhiza*.

Gradual degradation of the thylakoid system was observed in all the fronds. For example, wavy and dilated thylakoids were marked in mother fronds (Figure 8G), similar to *Lemna minor* exposed to Cd (Tkalec et al., 2008). The size of plastoglobuli decreases after 24 h of exposure. Thylakoids are pushed toward the chloroplast periphery in younger fronds, and starch grains occupy chloroplast volume. With prolonged exposure to Cd, chloroplasts are inflated and contain larger starch grains of irregular shape and structure. Plastoglobuli are small but numerous. In younger fronds, starch content increased with cadmium concentration and exposure duration, while in older fronds, starch content was increased in low Cd and decreased in higher concentrations (Figures 7A–F, 9D,E). The accumulation of starch grains resulted from an imbalance between photosynthate production and export. Therefore, the energy produced by metabolic reactions either reinforced the younger frond's resistance to cadmium stress or compensated for the reduced enzyme activity caused by the same Cd stress.

Although not directly analyzed in this study, possible differences in Cd uptake and accumulation in plants of different ages could influence photosynthetic activity, since Cd is a cumulative toxicant (Haider et al., 2021). It has been determined that *S. polyrhiza* can tolerate and accumulate Cd for 1 week even when exposed to 7 μg Cd ml^−1^(Sajwan and Ornes, 1994). The stage of plant development significantly affects physiological activity, especially its roots. It is known that Cd bonds to duckweed roots (Xue et al., 2018), affects the formation of turions by causing nutrient deficiency, and accumulates in mature fronds (Oláh et al., 2015). Cd accumulation depends on the number of binding sites in cells and the capacity to uptake Cd (Chen et al., 2021), and in *S. polyrhiza*, it is directly proportional to its exposure concentration and duration (Rolli et al., 2010). After several hours of exposure, we observed that *S. polyrhiza* rejected its roots to avoid further uptake. We have also observed the abscission phenomenon of colony disintegration upon exposure to Cd with almost immediate release of DFs from the maternal colony before reaching maturity (Figure 9 and Supplementary Figure 1). This avoidance strategy could also result in differences in Cd accumulation in young and mature fronds and needs to be taken into future consideration in explaining the sensitivity of the photosynthetic apparatus to Cd toxicity.

### Mothers vs. Daughters in Response to Cadmium

The oxygen-evolving complex was not affected by low Cd concentration in the mother fronds. Therefore, gross O_2_ production rate was not affected, and the supply of electrons was similar to the control fronds. However, with a higher PI_TOT_ decrease, both performance indices decreased, meaning that electrons had to be redirected toward alternative electron sinks. A shortage in NADPH could have been, thus induced, affecting the Calvin Benson cycle (Krupa et al., 1999; Hald et al., 2008). The increased starch grains observed in chloroplasts of MFs treated with the lowest cadmium concentration could also mean that the Calvin cycle was in a latent state. Since the dark O_2_ uptake rate was reduced, starch probably was not mobilized during the night, so decreased respiration rate could have possibly led to accumulation of reducing equivalents, changing the ratio of ATP/NADPH. The accumulation of reducing equivalents favored cyclic electron transport around PSI, which supplied additional ATP to chloroplasts by acidifying the thylakoid lumen (Huang et al., 2019, 2021).

On the other hand, acidification downregulates electron transport through the cytochrome *b*_6_*f* complex (Krieger-Liszkay et al., 2000; Shikanai, 2014). In this way, the regulation of homeostasis was achieved with additional ATP needed to maintain system stability. This alternative cyclic electron flow could have been mediated by either the proton gradient regulation 5 (PGR5)/PGR5-like 1 (PGRL1) pathway or by a NAD(P)H plastoquinone oxidoreductase (NDH) complex (Munekage, 2002; Shikanai, 2007), since both can reduce plastoquinone using ferredoxin as an electron donor. However, lumen acidification typically decreases the rate of plastoquinol oxidation, suggesting, according to Cardol et al. (2011), that this step is the bottleneck of photosynthesis.

The respiration rate in MFs was increased by increasing Cd concentration to C3, but the oxygen-evolving rate was decreased, and starch was mobilized, as shown in Figures 6, 9. Since NADPH deficit was probably partially compensated because of increased respiration, e.g., with activation of NADPH producing reactions by malic enzyme and glucose-6-phosphate dehydrogenase (Van Assche and Clijsters, 1990), the cyclic electron flow around PSI was activated to recompense increased needs for ATP. Furthermore, the mother fronds have numerous plastoglobules inside chloroplasts (Figure 7A). Their number and size were observed to decrease during 72 h of exposure to low Cd concentration (C1, Figure 7C). However, their number and size were unchanged in higher cadmium concentration (C3, Figure 7F). According to Austin et al. (2006) and Besagni and Kessler (2012), plastoglobuli participate in the synthesis, storage, and regeneration of tocopherols plastochromanol and phylloquinone, which they release to the thylakoid membrane through the attachment site. Plastoglobules also contain carotenoids. Carotenoids in the C1 treatment were probably recruited from plastoglobules and contributed to the detoxification of reactive oxygen. Besides, plastoglobules on their lipid monolayer surface contain specific proteins, many of which are involved in lipid metabolism like NADPH dehydrogenase (NDH), a known player in prenylquinone metabolism that reduces the PQ pool to PQH_2_ (Eugeni Piller et al., 2012; Rottet et al., 2016). Since enhanced synthesis of prenylquinones is implicated under oxidative stress, the reduced rate of plastoquinol oxidation by increased acidification in the C3 treatment could have been mitigated by the action of NADPH dehydrogenase. Thus, prenylquinone, namely plastoquinol, could have served as an electron carrier or more likely to reduce oxygen radicals O${}_{2}^{-}$ formed by electron redirection to O_2_ in the water-water cycle (Munekage et al., 2004; Miyake, 2010). Therefore, in C1 treatments of the mother fronds, carotenoids were sufficient for detoxification of ROS that originated around PSI, but in C3 other mechanisms had to be involved.

As for the younger daughter fronds (DF_2_s and GDFs), the faster respiration rate in both cadmium treatments did not result in starch mobilization, since starch accumulated with increase in Cd concentration (Figures 9F,G). Therefore, according to Vanlerberghe et al. (2020), faster respiration rate probably resulted in the activation of NADPH-producing reactions. However, the net exchange of oxygen in the light was 6-fold higher in C1 than in C3 because of the faster oxygen production rate in C1 and slower oxygen production rate in C3 (Figure 6). At the same time, the OEC complex was unaffected by both cadmium concentrations, meaning that, in both treatments, electrons were supplied as usual. However, electron transport from Q${}_{\text{A}}^{-}$ to PQ was reduced in the C1 treatment and was unaffected in the C3 treatment, which was reflected in PI_ABS_.

Similarly, based on PI_TOT_, the whole electron transport was more affected by C1 than by C3. So, why is electron flow to the PQ pool reduced in the C1 treatment? The increased oxygen production in C1 treatment could be linked to increased specific energy fluxes per active PSII (ABS/RC, TR_0_/RC, and DI_0_/RC), activating cyclic electron flow around PSII (CEF-II) to enhance the electron flux through PSII and oxidize the PQ pool. CEF-II, in turn, could activate cyclic electron flow around PSI, accelerating the change in pH (or acidification), leading to accumulation of powerful reductant P700^+^ and contributing to the dissipation of excess energy as heat to protect PSI (Miyake and Okamura, 2003; Kadota et al., 2019). The decrease in electron transport from Q${}_{\text{A}}^{-}$ to PQ is responsible for the subsequent reduction of PI_ABS_, while electron transport from Q${}_{\text{A}}^{-}$ to final electron acceptors was responsible for reducing PI_TOT_. Unaffected OEC complex means that O_2_ was produced as usual, confirmed with no net change in O_2_ fluxes in the light. However, due to some RCs' inactivation, increased absorption, and trapping per active RCs, more O_2_ was produced. Phenomenological energy fluxes per cross-section were no different from the control values, except for RE_0_/CS_0_, which was decreased significantly. On the other hand, in the C3 cadmium treatment, ABS, TR_0_, and ET_0_ per active RC were similar to the control values, while the density of active RCs per CS was increased. All phenomenological energy fluxes, except for RE_0_, increased, indicating increased photosynthetic capacity per CS. These increased fluxes had a stimulating effect that stopped PI_ABS_ decrease. However, the energy was lost more (DI_0_/CS_0_ and DI_0_/CS_m_), reducing the available energy for photosynthesis.

## Conclusions

Reduced respiration rate, shortage of NADPH, and high starch in older mother fronds exposed to low Cd resulted in accumulated reducing equivalents favoring cyclic electron flow around PSI, allowing the supply of additional ATP to maintain system stability. When exposed to higher Cd concentration, increased respiration rate and mobilized starch compensated the NADPH deficit and activated the alternative cyclic electron flow around PSI and water-water cycle. Plastoquinol had to reduce resulting radicals, thus reducing the available energy for photosynthesis and causing more significant damage.

Faster respiration and mobilization of starch in younger fronds exposed to low Cd concentration resulted in activation of NADPH producing reactions. At the same time, because of unaffected OEC but reduced electron transport rate, electron flux through PSII was enhanced by the cyclic electron flow around PSII and PSI. At higher Cd concentration, electron transport rate was unaffected because of increased phenomenological fluxes that converted inactive PSII into a functional state and stopped the decrease in photosynthetic efficiency, causing less damage.

In conclusion, the ultrastructural observation and chlorophyll fluorescence measurements showed that exposure to cadmium causes differences in photosynthetic efficiency, which are age-related (Figure 9H). The younger fronds showed less loss of energy connectivity than the older fronds and less inhibition of electron flow on the acceptor side of PSII. This inhibition was increased with increase in Cd concentration. Their potential to sustain photochemical events at higher cadmium concentrations as opposed to the older maternal fronds allows them to cope more efficiently with cadmium-induced stress. Therefore, the age of plants is an important parameter to consider when assessing the photosynthetic and photoprotective abilities of plants in response to heavy metal stress. Overall, the adverse effects of Cd toxicity were concentration- and exposure duration-dependent, since ultrastructural changes were visible very early after exposure to the lowest cadmium concentration in the all fronds. Placing age-related changes in the time frame of Cd action provides valuable information on the toxicological profile of Cd in terms of both concentration and duration of exposure. This research contributes to a more accurate and representative characterization of plant responses to heavy metal stress.

## Data Availability Statement

The raw data supporting the conclusions of this article will be made available by the authors, without undue reservation.

## Author Contributions

VP and JA were responsible for conceptualization, methodology, investigation, formal analysis, writing of the original draft, and supervision. LD performed the experiment, data collection, and laboratory analysis. GZ and VC provided leadership, resources, and critical review and editing of the initial draft. All authors have read and approved the final version of the manuscript.

## Funding

This study was supported by the Department of Biology, Josip Juraj Strossmayer University of Osijek Research Block Grant Program.

## Conflict of Interest

The authors declare that the research was conducted in the absence of any commercial or financial relationships that could be construed as a potential conflict of interest.

## Publisher's Note

All claims expressed in this article are solely those of the authors and do not necessarily represent those of their affiliated organizations, or those of the publisher, the editors and the reviewers. Any product that may be evaluated in this article, or claim that may be made by its manufacturer, is not guaranteed or endorsed by the publisher.

## Abbreviations

ABS: absorbed photon flux
ABS/RC: average absorbed photon flux per PSII reaction center
ABS/CS: absorbed photon flux per excited cross-section of PSII (or also apparent antenna size)
Car: carotenoid
CEF: cyclic electron flow
Chl: chlorophyll
CS: a cross-section of PSII
DF: daughter frond
DI_0_/ABS: quantum yield of energy dissipation in PSII antenna
DI_0_/RC: the flux of energy dissipated per active RC
ET_0_/RC: electron transport flux from $Q_{A}^{-}$ to PQ per active PSII
F_0_: initial fluorescence value
F_300μ*s*_: fluorescence value at 300 μs
F_m_: maximal fluorescence intensity
F_V_: maximum variable fluorescence
GDF: granddaughter frond
M_0_: initial slope (in ms^−1^) of O-J
MF: mother frond
OEC: oxygen-evolving complex
PCA: principal component analysis
PI_ABS_: performance index (potential) for energy conservation from exciton to reduction of intersystem electron acceptors
PI_TOT_: performance index (potential) for energy conservation from exciton to reduction of PSI end acceptors
Q_A_ and Q_B_: primary and secondary quinone electron acceptors
PQ: the pool of free plastoquinone behind the PSII reaction center
PQH_2_: plastoquinol
PSI: photosystem I
PSII: photosystem II
RC: total number of PSII active reaction centers
RE_0_/RC: electron transport flux from $Q_{A}^{-}\;$to final PSI acceptors per active PSII
RE_0_/CS: electron transport flux from $Q_{A}^{-}$ to final PSI acceptors per cross-section of PSII
S_m_,: the normalized area between the OJIP curve and the line *F*_*m*_ which is a proxy of the number of electron carriers per electron transport chain
TEM: transmission electron microscope
TR_0_/RC: maximum trapped exciton flux per active PSII
TR_0_/CS: maximum trapped exciton flux per cross-section
V_I_: relative variable fluorescence at 30 ms (I-step)
V_J_: relative variable fluorescence at 2 ms (J-step)
V_K_: relative variable fluorescence at 300 μs (K-step)
V_t_: relative variable fluorescence at time t
ΔV_IP_: I-P normalized differential induction curves
ΔV_OJ_: O-J normalized differential induction curves
ΔV_OK_: O-K normalized differential induction curves
ΔV_OP_: O-P normalized differential induction curves
δR_0_: efficiency with which an electron from Q_B_ is transferred to final PSI acceptors
ϕE_0_: quantum yield of electron transport from $Q_{A}^{-}$ to PQ
ϕP_0_: maximum quantum yield of primary PSII photochemistry
ϕR_0_: quantum yield of electron transport from $Q_{A}^{-}$ to final PSI acceptors
ψE_0_: efficiency with which a PSII trapped electron is transferred from Q_A_ to Q_B_
ψR_0_: efficiency with which a PSII trapped electron is transferred to final PSI acceptors.
