# Supplementary material for: Time Course of Age-Linked Changes in Photosynthetic Efficiency of Spirodela polyrhiza Exposed to Cadmium
Source: Front Plant Sci. 2022 May 25;13:872793. doi: 10.3389/fpls.2022.872793 (PMC9175006; doi:10.3389/fpls.2022.872793)
Supplement: Supplementary file 1 [file Data_Sheet_1.DOCX]

Supplementary Material

## Supplementary Figures and Tables


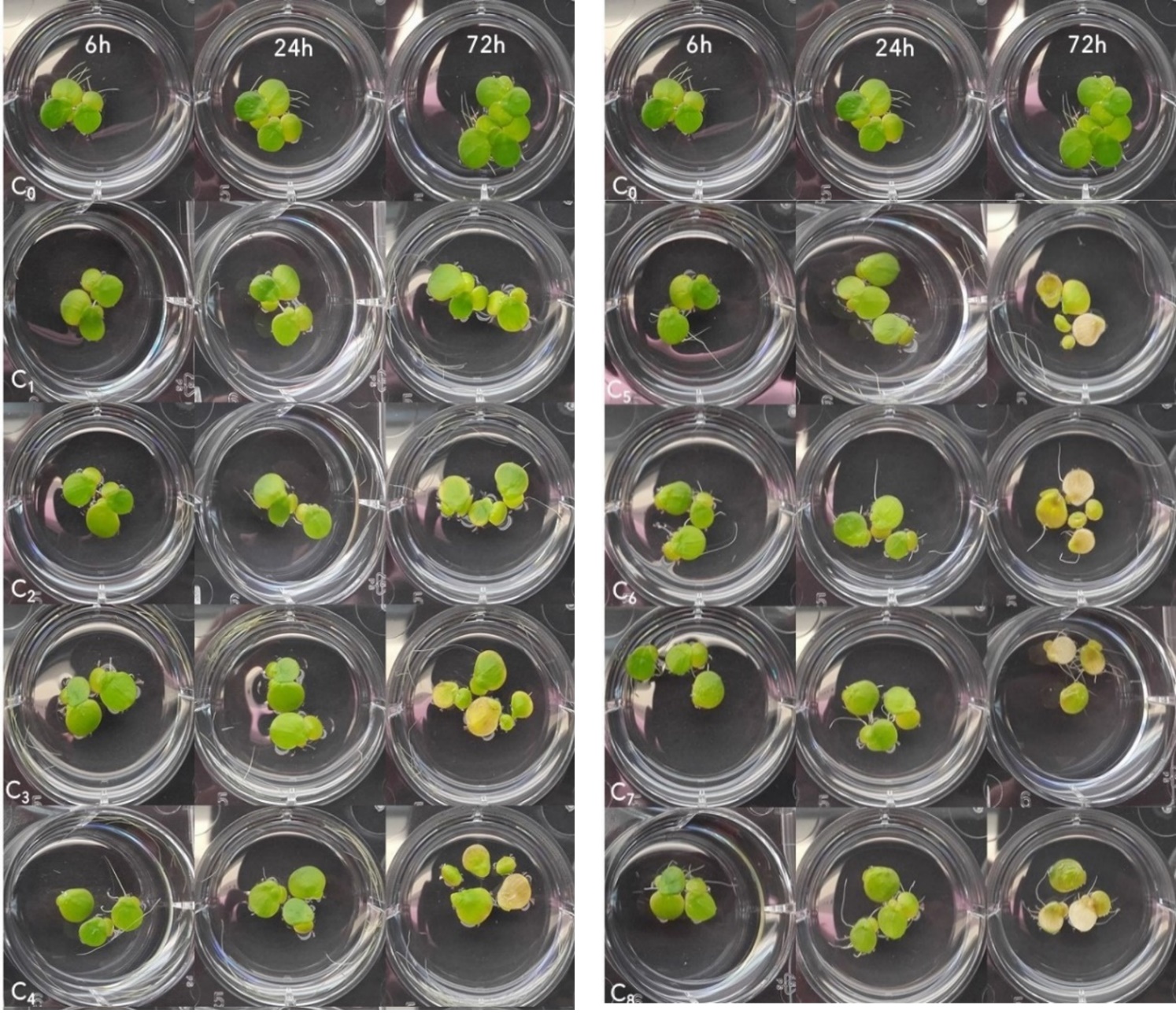


**Supplementary Material Figure 1.** Visual symptoms of *S. polyrhiza* in C0 (control, StMo medium) and after 6, 24, and 72 h of exposure to Cd treatments (7 μM - C1 to 890 μM - C8 concentration).


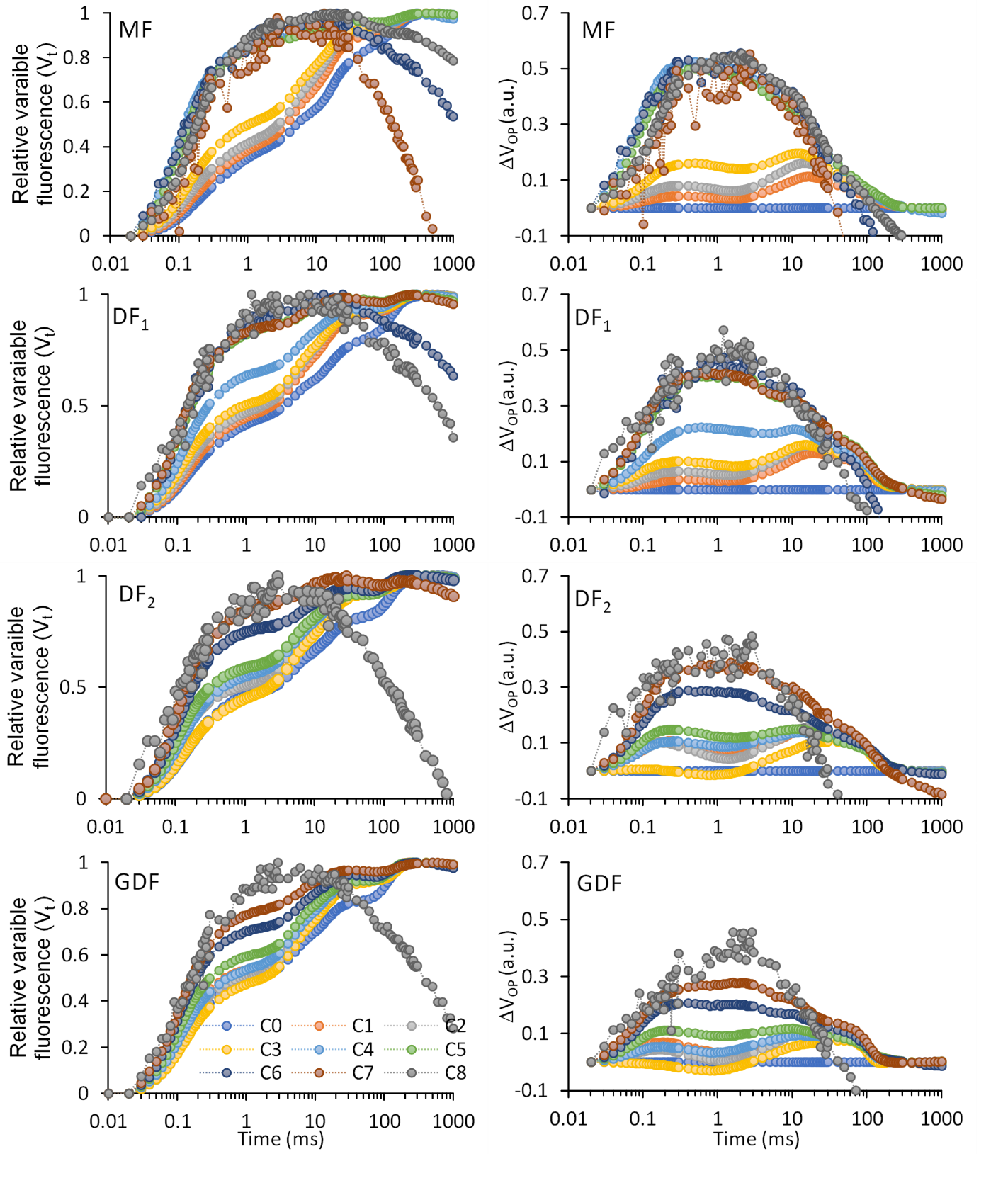


**Supplementary Material Figure 2.** Relative variable fluorescence ( $V_{t}=\left[ \left( F_{t}-F_{0} \right)/\left( F_{P}-F_{0} \right) \right]$) of different chlorophyll *a* fluorescence transient obtained from dark-adapted *S. polyrhiza* plants (MF – mother frond; DF_1_ – first daughter frond; DF_2_ - second daughter frond; GDF - granddaughter frond) in control (C0, StMo medium) and after 72 h of exposure to Cd treatments (7 μM - C1 to 890 μM - C8 concentration); O-P normalized differential induction curves (${\Delta V_{OP}=\left[ \left( F_{t}-F_{0} \right)/\left( F_{P}-F_{0} \right) \right]}_{treatment}-\left[ \left( F_{t}-F_{0} \right)/\left( F_{P}-F_{0} \right) \right]_{control}$). The signals are plotted on a logarithmic scale. Each curve is an average of at least six replicates.


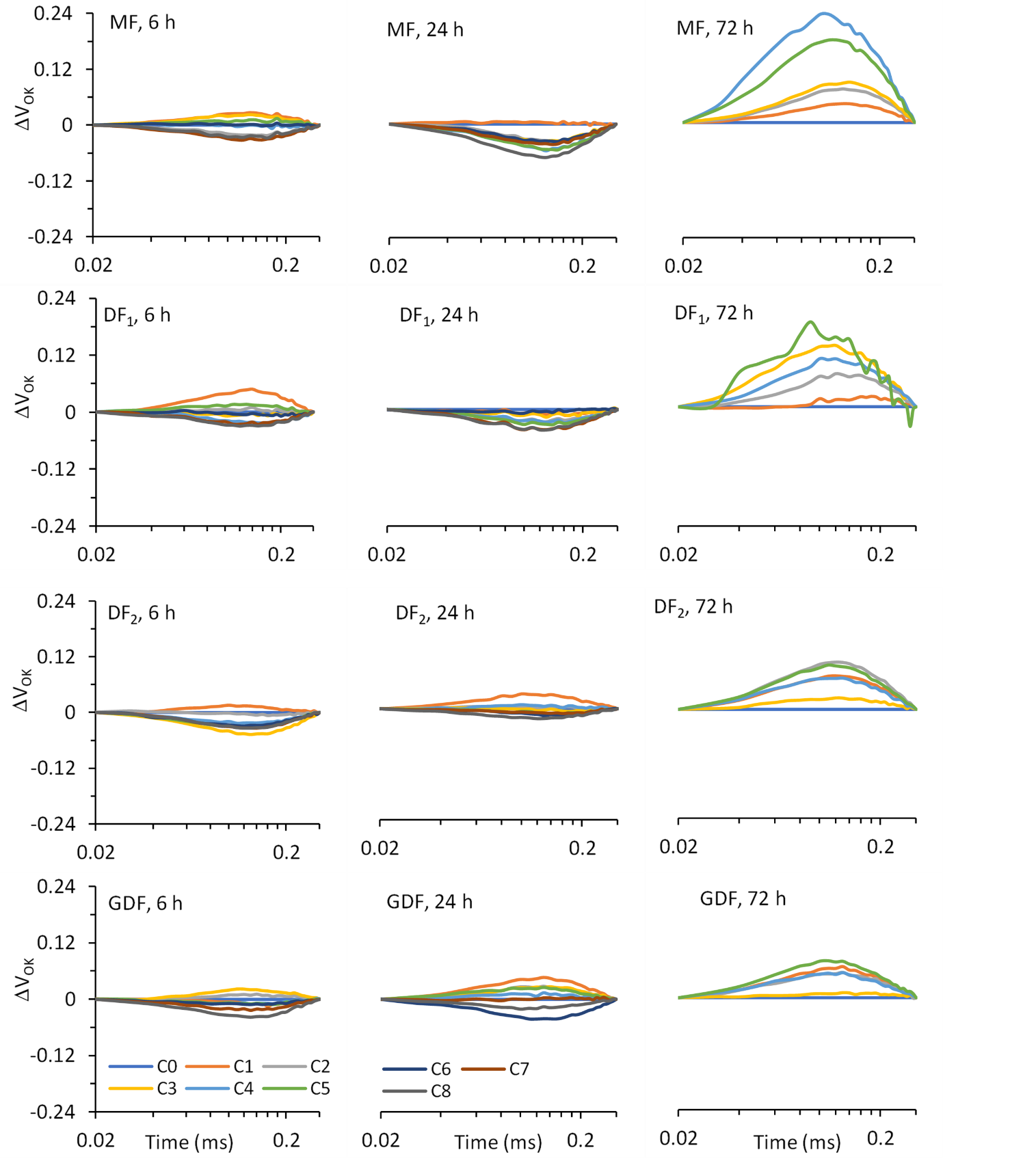


**Supplementary Material Figure 3.** Differential curves of chlorophyll *a* fluorescence transient normalized between O-K steps. ${\Delta V_{OK}=\left[ \left( F_{t}-F_{0} \right)/\left( F_{K}-F_{0} \right) \right]}_{treatment}-\left[ \left( F_{t}-F_{0} \right)/\left( F_{K}-F_{0} \right) \right]_{control}$ of *S. polyrhiza* after 6, 24, and 72 h of exposure to Cd (C1-C8) in mother fronds (MF), first daughter (DF_1_), second daughter (DF_2_), and granddaughter fronds (GDF). Each curve is an average of six replicates.


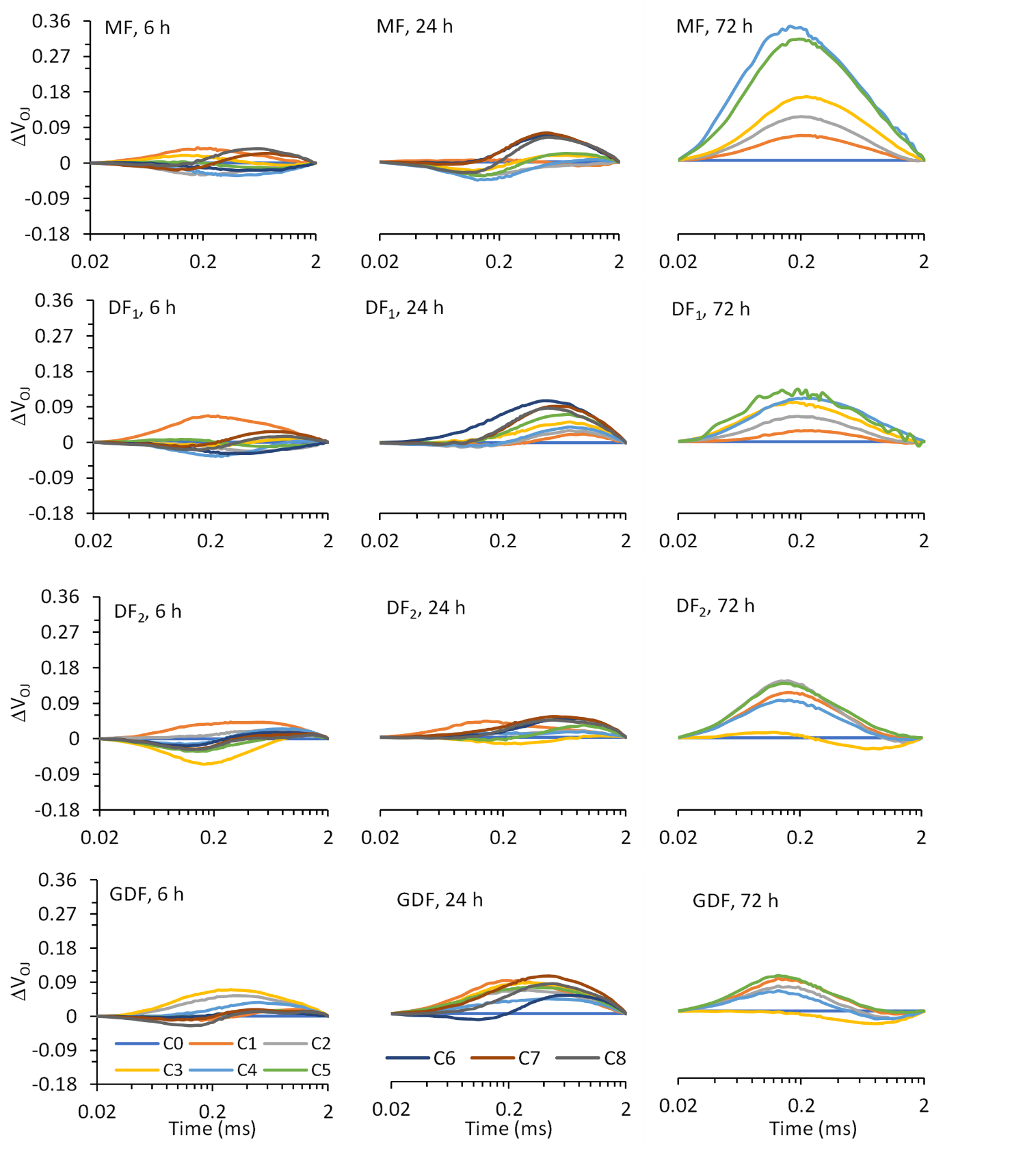


**Supplementary Material Figure 4.** Differential curves of chlorophyll *a* fluorescence transient normalized between O-J steps. ${\Delta V_{OJ}=\left[ \left( F_{t}-F_{O} \right)/\left( F_{J}-F_{O} \right) \right]}_{treatment}-\left[ \left( F_{t}-F_{O} \right)/\left( F_{J}-F_{O} \right) \right]_{control}$ of *S. polyrhiza* after 6, 24, and 72 h of exposure to Cd (C1-C8) in mother fronds (MF), first daughter (DF_1_), second daughter (DF_2_), and granddaughter fronds (GDF). Each curve is an average of six replicates.

**
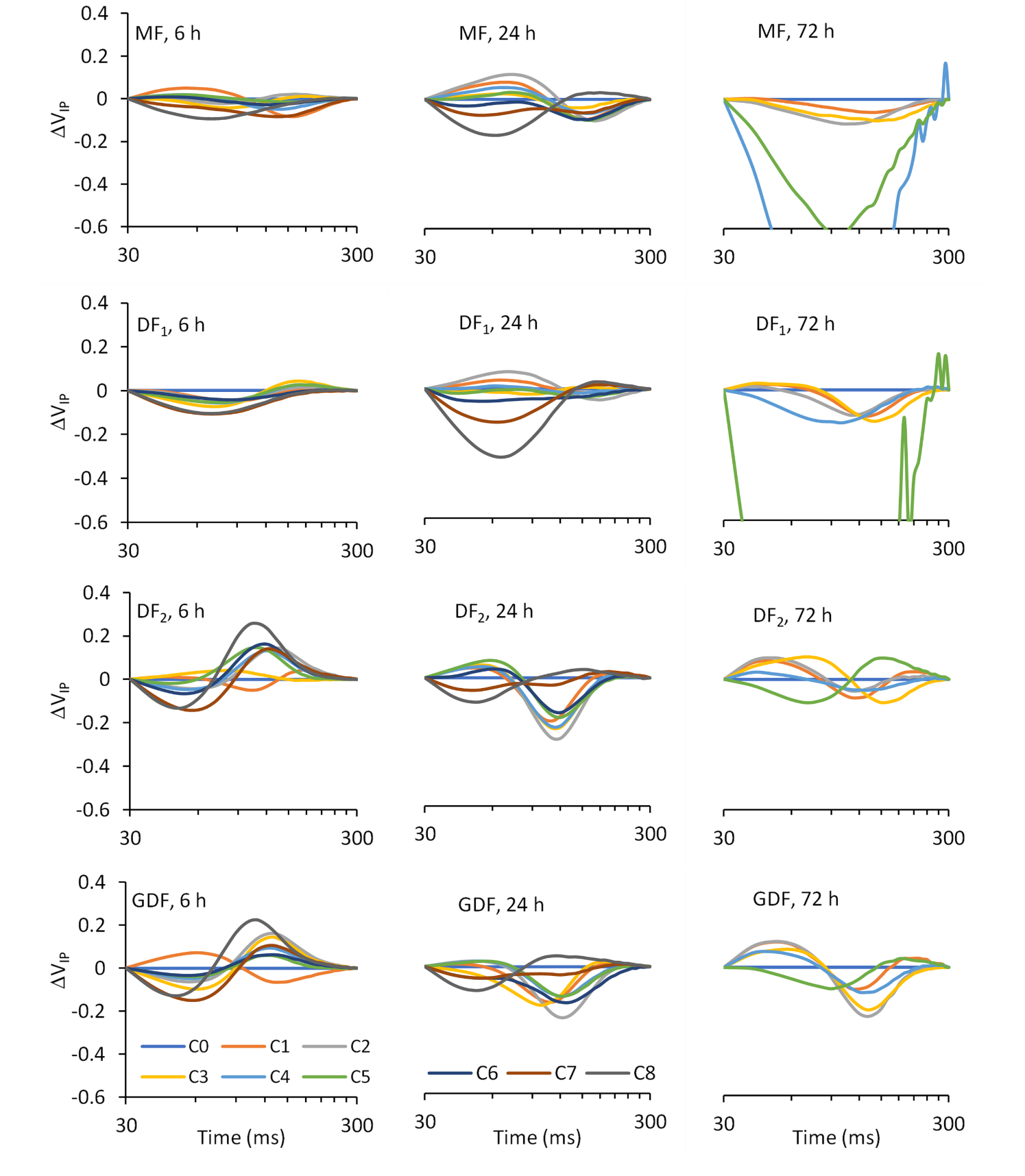
**

**Supplementary Material Figure 5.** Differential curves of chlorophyll *a* fluorescence transient normalized between I-P steps. ${\Delta V_{IP}=\left[ \left( F_{t}-F_{I} \right)/\left( F_{P}-F_{I} \right) \right]}_{treatment}-\left[ \left( F_{t}-F_{I} \right)/\left( F_{P}-F_{I} \right) \right]_{control}$ of *S. polyrhiza* after 6, 24, and 72 h of exposure to Cd (C1-C8) in mother fronds (MF), first daughter (DF_1_), second daughter (DF_2_), and granddaughter fronds (GDF). Each curve is an average of six replicates.

**Supplementary Material Table 1.** Summary of measured fast chlorophyll *a* fluorescence transient and calculated OJIP test parameters where O stands for origin (the minimum fluorescence F_0_), J and I are intermediate levels at 2 ms and 30 ms, and P is a peak at F_m_ or maximal fluorescence. PSI – photosystem I, PSII – photosystem II, RC – total number of PSII active reaction centers, CS – a cross-section of PSII, and Q_A_ – plastoquinone A, Q_A_ –plastoquinone B, and PQ – the pool of free plastoquinone behind the PSII reaction center.

| Parameter | Description |
| --- | --- |
| *Technical fluorescence parameters* | |
| $F_{0}\cong F_{0.02ms}$  $F_{m}$  $F_{V}=F_{m}-F_{0}$  $V_{t}={(F_{t}-F_{0})}/{(F_{m}-F_{0})}$  $M_{0}={({\Delta V}/{\Delta t)}}_{0}$  $\approx4(F_{0.3ms}-F_{0.02ms})/F_{V}$  $S_{m}=Area/F_{V}$ | Initial fluorescence value (at 20 μs)  Maximal fluorescence value (at 30 ms)  Maximum variable fluorescence  Relative variable fluorescence  Initial slope (in ms^-1^) of the O-J  The normalized area between the OJIP curve and the line $F_{m}$, which is a proxy of the number of electron carriers per electron transport chain |
| *Energy fluxes* | |
| ABS  TR  DI  ET  RE | Adsorbed photon flux (rate of photon absorption by total PSII antenna  Trapped exciton flux (rate of exciton trapping by all PSII RCs leading to Q_A_ reduction)  Dissipated energy flux (rate of energy dissipation in all PSII other than trapping)  Electron transport flux from Q_A_ to Q_B_  Electron transport flux until PSI acceptors |
| *Efficiencies and quantum yield* | |
| ${\psi E}_{0}= {ET}_{0}/{TR}_{0}$  ${\psi R}_{0}= {RE}_{0}/{TR}_{0}$  ${\delta R}_{0}= {\psi R}_{0}/{\psi E}_{0}$  ${\varphi P}_{0}= {TR}_{0}/ABS$  ${\varphi E}_{0}= {\varphi P}_{0}\times{\psi E}_{0}$  ${\varphi R}_{0}= {\varphi P}_{0}\times{\psi R}_{0}$  ${DI}_{0}/ABS$ | Efficiency with which a PSII trapped electron is transferred from Q_A_ to Q_B_  Efficiency with which a PSII trapped electron is transferred to final PSI acceptors  Efficiency with which an electron from Q_B_ (PQH_2_) is transferred to final PSI acceptors  Maximum quantum yield of primary PSII photochemistry  Quantum yield of electron transport from $Q_{A}^{-}$ to PQ  Quantum yield of electron transport from $Q_{A}^{-}$ to final PSI acceptors  Quantum yield of energy dissipation in PSII antenna |
| *Specific energy fluxes* | |
| $ABS/RC$  ${TR}_{0}/RC$  ${ET}_{0}/RC$  ${RE}_{0}/RC$  ${DI}_{0}/RC$ | Average absorbed photon flux per PSII reaction center (apparent antenna size of an active RC)  Maximum trapped exciton flux per active PSII  Electron transport flux from $Q_{A}^{-}$ to PQ per active PSII  Electron transport flux from $Q_{A}^{-}$to final PSI acceptors per active PSII  The flux of energy dissipated (other than trapping) per active PSII |
| *Phenomenological energy fluxes (per excited cross-section CS)* | |
| $ABS/{CS}_{0}\approx F_{0}$and $ABS/{CS}_{m}\approx F_{m}$  ${TR}_{0}/CS$  ${ET}_{0}/CS$  ${RE}_{0}/CS$ | Absorbed photon flux per excited cross-section of PSII (or also apparent antenna size)  Maximum trapped exciton flux per cross-section  Electron transport flux from $Q_{A}^{-}$ to PQ per cross-section of PSII  Electron transport flux from $Q_{A}^{-}$ to final PSI acceptors per cross-section of PSII |
| *Performance indexes (of PSII and specific electron transport reactions)* | |
| ${PI}_{ABS}= {RC}/{ABS\times}\left[ {\varphi P}_{0}/\left( 1-{\varphi P}_{0} \right) \right] \times\left[ {\psi E}_{0}/\left( 1-{\psi E}_{0} \right) \right]$  ${PI}_{ABS}^{total}={PI}_{ABS}\times\left[ {\delta R}_{0}/\left( 1-{\delta R}_{0} \right) \right]$ | Performance index (potential) for energy conservation from exciton to the reduction of intersystem electron acceptors  Performance index (potential) for energy conservation from exciton to the reduction of PSI end acceptors |
